# Reduced life cycle climate impact from manure through catalytic methane conversion and carbon dioxide removal

**DOI:** 10.1038/s41598-025-27609-2

**Published:** 2025-12-10

**Authors:** Emma Bromark, Devesh Sathya Sri Sairam Sirigina, Shareq Mohd Nazir, Pernilla Tidåker, Åke Nordberg, Per-Anders Hansson

**Affiliations:** 1 Department of Energy and Technology, Swedish University of Agricultural Sciences, Lennart Hjelms väg 9, Uppsala, 756 51 Sweden; 2 Department of Chemical Engineering, KTH Royal Institute of Technology, Teknikringen 42, Stockholm, 11428 Sweden

**Keywords:** GGR, CH_4_, Methane emissions, LCA, Manure management, Climate change, Engineering, Environmental impact

## Abstract

Agri-food systems constitute around one-third of global greenhouse gas (GHG) emissions, with roughly half consisting of non-CO_2_ GHGs, mainly methane (CH_4_) and nitrous oxide (N_2_O). Methods and technologies to mitigate non-CO_2_ GHGs are currently limited, which is a reason for agriculture being categorised as a hard-to-abate sector. This study examines mitigation of GHG emissions from manure storage headspace through oxidisation of CH_4_ emissions at low concentrations, using a thermal catalytic process with and without subsequent CO_2_ capture and storage (CCS). The technology is studied using a combination of process modelling and life cycle assessment at four CH_4_ concentrations: 300, 1000, 3000 and 10,000 ppmv. The primary energy demand and net climate effect were evaluated, reaching a net climate effect of +0.10, -0.77, -0.91 and -0.97 g CO_2_-eq emitted/g CO_2_ mitigated, respectively. The wide range of results is mainly influenced by the process energy demand being strongly correlated to the CH_4_ concentration. The sensitivity analysis shows that a net negative climate effect can also be achieved at 300 ppmv with access to low emission energy sources. Coupling CCS worsens the net climate effect of the system at all studied CH_4_ concentrations, mainly due to the additional energy demand for CO_2_ separation.

**Supplementary Information:**

The online version contains supplementary material available at 10.1038/s41598-025-27609-2.

## Introduction

The increase in greenhouse gas (GHG) emissions and climate scenario modelling shows the need for rapidly regressing GHG emissions through the parallel deployment of mitigation efforts and negative emissions across all of sectors^1^. However, some sectors are regarded as more challenging to handle than others. The GHG emissions from the agricultural sector are considered hard-to-abate, as they are predominantly not tied to energy use but rather originate from animals, soils and manure; thus, a renewable energy transition will not impact these emissions^2^. The global climate impact from agri-food systems amounted to 16 Gt carbon dioxide equivalents (CO_2_-eq) in 2022, with over half caused by non-CO_2_ GHGs, mainly methane (CH_4_) and nitrous oxide (N_2_O)^3^. The low concentrations (typically < 1%-vol) and dispersed nature of the emissions make them difficult to quantify, target and reduce^4,5^. This indicates a need for different types of mitigation strategies for GHG emissions in agriculture and that a future net-zero or net-negative emissions balance would require residual emissions being offset through greenhouse gas removal (GGR)^6^. GGR can be achieved through natural (biological) processes as well as engineered (technical) systems, i.e. greenhouse gas removal technologies (GGRTs)^7^. A rather versatile portfolio of GGRTs has been presented, with the majority involving carbon dioxide removal (CDR)^8^. However, second only to CO_2_, CH_4_ is such a potent of a GHG that it contributes to 25% of total radiative forcing (RF), despite its atmospheric concentration being only 0.5% that of CO_2_^9^. Due to its high RF and short atmospheric residence time (approximately 12 years), rapid reductions in CH_4_ emissions have great potential to impact global warming in the short term^10^. Despite there being a strong case for CH_4_ mitigation, it remains poorly covered in climate policy, although it has received increased attention in recent years^11^.

A strategy to evade the warming effect of a high GWP gas such as CH_4_ is to convert it into a lower GWP gas, such as CO_2_^8^. Such research has been put forward, with suggested applications both for the fossil fuel industry^12^ and ambient air e.g.^13–15^, although these are at a low technological readiness level^16^. CH_4_ gradually oxidizes in the atmosphere over time, but this reaction can be initiated by, for example, introducing a catalyst and/or energy addition via light, heat or electricity^17^. For example, de Richter et al.^15^ used photocatalysts to convert CH_4_ from ambient air, a technology that could be applied to mitigate methane from concentrated sources. Ming et al.^14^ discussed several technologies to remove methane from atmosphere, such as enhancing hydroxyl radicals, generation of chlorine atoms, methanotrophic bacteria in bioreactors. CH_4_ removal from ambient air would require large air flows due to the low concentration^18^. There are published studies that focus mainly on the catalyst development^19–21^ for treating low-concentration methane emissions but less on the process technology or assessed using a wider systems perspective. Sirigina et al.^22^ proposed process concepts based on thermal-catalytic oxidation of methane at low concentrations, which forms the basis for this study.

Over half of the global CH_4_ emissions originates from agrifood systems^23^. The scientific focus of climate intervention in the agricultural sector has so far been on CH_4_ emissions management approaches rather than removal measures. None of the previously mentioned studies^12–23^ have evaluated agricultural applications. Many CH_4_ emissions arise from large poorly constrained areas, such as organic soils, rice fields and wetlands^4,24^. Since the higher the concentration of CH_4_, the easier it is to get the CH_4_ conversion reaction running^22^, it is preferable to identify a CH_4_ point source to increase process efficiency and decrease component size^25^. Possible alternatives of concentrated emission sources include manure storage or stables for ruminants^26^. Livestock cause considerable CH_4_ emissions originating from enteric fermentation (~ 70%) and manure (~ 30%)^23^. GHG emissions from manure is the second largest emission source from farms^23^. During manure storage, organic matter in the manure starts to decompose, forming CO_2_ under aerobic conditions and CH_4_ through anaerobic reactions^27^. The rate of emissions depends on a number of factors, such as organic matter, temperature, moisture and pH^28^, and a future warmer climate would increase CH_4_ emissions from manure^29^. Manure degradation can be partially regulated by lowering the pH of the manure through cooling, increasing acidity or covering the source^29–31^. The manure could also be subjected to anaerobic digestion, which allows the CH_4_ to form under controlled circumstances where it can be collected and utilised for its energy content^32^. Despite this being an efficient utilisation of the waste and lowered climate impact, only around 5% of manure is digested in Sweden, one barrier being the high investment cost of farm-based biogas plants^33^. A potential approach that has not yet been explored is to treat the air from the manure storage space on-site by oxidising the CH_4_ in a reactor containing a catalytic material to facilitate the reaction, as was recently proposed by Sirigina, et al.^22^. The approach of this system is to convert the CH_4_ as soon as it is emitted to avoid its warming effect, and the conditions allows adaption to the CH_4_ concentrations that are typical to agricultural sources. The resulting CO_2_ from the oxidation reaction is biogenic in nature, which can be captured and stored. The most common technologies to capture CO_2_ are chemical absorption and adsorption^34^. Geological storage is necessary to achieve long-term removal of the CO_2_ from the atmosphere^35^.

To achieve sustainable and credible GGR, the applied technologies must result in a net negative emission balance over the system’s lifecycle and long-term storage reliability of the removed emissions^36^. There is a growing number of studies of CDR technologies which emphasise the importance of the life cycle assessment (LCA) methodology to evaluate its effectiveness in delivering negative emissions, suggesting clear variance in effectiveness across technologies (see e.g. review by Rueda, et al.^1^). However, the life-cycle perspective of GGR through the conversion of CH_4_ emissions emerging from biogenic sources is currently in its infancy with only one scientific publication, on photocatalytic oxidation of CH_4_^37^. There is also a need for further discussion about scenarios where GGR targeting CH_4_ emissions can be considered as a greenhouse gas removal or mitigation strategy. This study aims to contribute with an evaluation of thermal catalytic oxidation as an option for reducing CH_4_ emitted from manure storage using a life cycle perspective. The objective of the study was to determine the energy demand and climate efficiency of the proposed technology. Furthermore, the impact of releasing the exhaust gas to the atmosphere after the conversion process was compared with subsequent capture and storage of the CO_2_ from the exhaust gas. The study attempted to identify energy and climate related hotspots in the process to provide guidance for future system development.

## Methodology

This study investigated a process technology for thermal catalytic treatment of the CH_4_ emitted from manure storage and subsequent CO_2_ capture and storage (CCS). The approach was to compare a reference state of conventional unabated manure storage emissions to a scenario where the manure storage space is integrated with the suggested technology:

- Scenario A: *CH*_*4*_*-conversion*. GHG emissions from manure storage headspace is treated through catalytic CH_4_ oxidation and the exhaust gas is released into the atmosphere.
- Scenario B: *Co-removal*. GHG emissions from manure storage headspace is treated through catalytic CH_4_ oxidation followed by capture and storage of the CO_2_ in the outlet gas.

The scenarios are explained in greater detail below. The study was performed through a combination of process modelling in Aspen Plus V12 and LCA. Both the LCA model and the process model relied largely on generic literature data and are not meant to represent a specific installation or determine an optimal system of production, but rather to provide a representative example based on currently available data.

### Life cycle assessment

The study covered each process step involved, from manure storage to the suggested catalytic treatment until the exhaust gas is released or treated for CO_2_ capture for storage after the process. Processes occurring upstream of manure storage were excluded as they remain unaffected throughout the scenarios. The system boundary considered was cradle-to-grave, meaning the study included emissions from the entire life cycle of the technical appliances, including the necessary energy and raw materials, transport and processing, manufacturing of machinery, infrastructure and facilities, as well as its end of life. The life cycle inventory (LCI) was established through data collection from the literature and by utilizing the process parameters obtained from process models developed in Aspen (Sect. 2.3), with the resulting data used for the life cycle impact assessment. LCI data on a European level was primarily used and, if the corresponding data was not available, on a global level. As a final option, country-specific LCI datasets were used. The data was compiled over the three life cycle phases: manufacturing (raw material extraction and processing), operation (plant in operation), and end of life (material waste management). The climate impact and primary energy demand (PED) for the raw material for the process components manufacturing and catalyst was based on data from Ecoinvent v. 3.9.1. End of life for the materials required were included based on Ecoinvent^38^ data.

The functional unit provides a quantitative measure of the function(s) of the assessed process and serves as a basis for comparison^39^. For GGRTs, a functional unit that reflects the function of lowered global warming is encouraged, allowing comparison between different GGR options^36^. Therefore, we used the functional unit (FU) ‘grams (g) of CO_2_-equivalents (CO_2_-eq) mitigated’. The resulting impacts from the LCA is related to the functional unit in accordance with Eq. (1):

1 $$\:impact\:score=\frac{enviromental\:impact}{FU}$$

The impact categories considered are primary energy demand (PED) in kilojoules (kJ) (Eq. 2) and net climate effect (CE), defined as g CO_2_-eq emitted relative to the CO_2_-eq mitigated (Eq. 3):

2 $$\:PED=ED*PEF$$

3 $$\:net\:CE=\frac{{e}_{emitted}-{e}_{mitigated}}{{e}_{mitigated}}.\:$$

*ED* denotes energy demand, *PEF* the primary energy factor and *e* GHG emissions in g CO_2_-eq. By this definition, a negative value of the net CE denotes lower system emissions than mitigated emission, whereas a positive value of the net CE is to be interpreted as the system causing more GHG emissions than is being mitigated. As the GHGs emitted from manure are of biological origin, their removal would be categorized as negative emissions, provided that the total removal of GHG emissions is larger than the total GHG emissions emitted to the atmosphere by the processes required for GGR^36^. Compared to the reference state, oxidising CH_4_ as soon as it is emitted will help eliminate the large RF which is otherwise exerted during its residence time in the atmosphere. This was shown as an arithmetically negative contribution to the net CE, although the carbon atom is still present in the atmosphere in the CH_4_ conversion case. However, the term mitigated was chosen as the assumption that we are in fact removing or mitigating emissions needs a broader system level discussion, which is outside the scope of this article.

GWP100 was used as the conversion metric to assess climate impact from GHG emissions as is standard practice in LCA. However, with CH_4_ being such a central component of this study, the metric choice was subjected to sensitivity analysis to highlight its implications, by replacing GWP100 with GWP20 and GWP500. GWP values as defined in AR6 were used^40^. To allow this, disaggregated GHG emission data was used when possible.

The energy used for operation was solely in the form of electricity. The electricity source was assumed to be a European mix with an emission factor of 293 g CO_2_-eq/kWh^41^ and a primary energy factor of 2.3^42^. This was subjected to sensitivity analysis, exchanging the electricity source for natural gas power with 436 g CO_2_-eq/kWh^43^ and a primary energy factor of 2.0, and for a more renewable mix represented by the Nordic consumption mix (approximately 50% hydropower, 20% nuclear power, 15% wind and solar power and 15% combined heat and power) with 93.2 g CO_2_-eq/kWh^44^ and primary energy factor of 1.7^45^. Data was chosen to cover life-cycle emissions from electricity generation.

### System description

Livestock slurry was assumed to be stored on a farm in covered manure storage with a capacity of 5000 m^3^, sufficient for manure from around 200 dairy cows including a grazing period of 3 months^46^. The manure was stored for up to 9 months before being spread on nearby fields as fertilizer. Biological processes degrading the organic matter in the manure form emissions of gases and heat. Based on IPCC guideline data^47^ for the yearly average CH_4_ emissions, the CH_4_ emission rate from the manure was determined to be 0.390 mg of CH_4_ (10.5 mg CO_2_-eq) per second. In the reference state, the GHG emissions were released to the atmosphere without any intervention. For this analysis we chose to model four different concentrations of CH_4_ (300, 1000, 3000, and 10,000 ppmv), representing conditions which cause low to high CH_4_ emissions (see Table S1 for context). This was achieved by adjusting the dilution of CH_4_ in four different airflows (6890, 2067, 689 and 207 m^3^/h), respectively. This interval gives information on how the emissions rate relates to the size of the process plant and covers a relevant range of operating conditions. We further assume the share of aerobic vs. anaerobic degradation of organic matter in the manure storage was such that the GHG emissions consisted of CO_2_ and CH_4_ on a 40:60 molar% basis based on Grant, et al.^48^. The CO_2_ originating from the manure degeneration (0.71 g/s) entered the process alongside the CO_2_ in ambient air, which was set to 417 ppmv. Details can be found in the Supplementary material (SM), Tables S3 and S4.

**Fig. 1:**
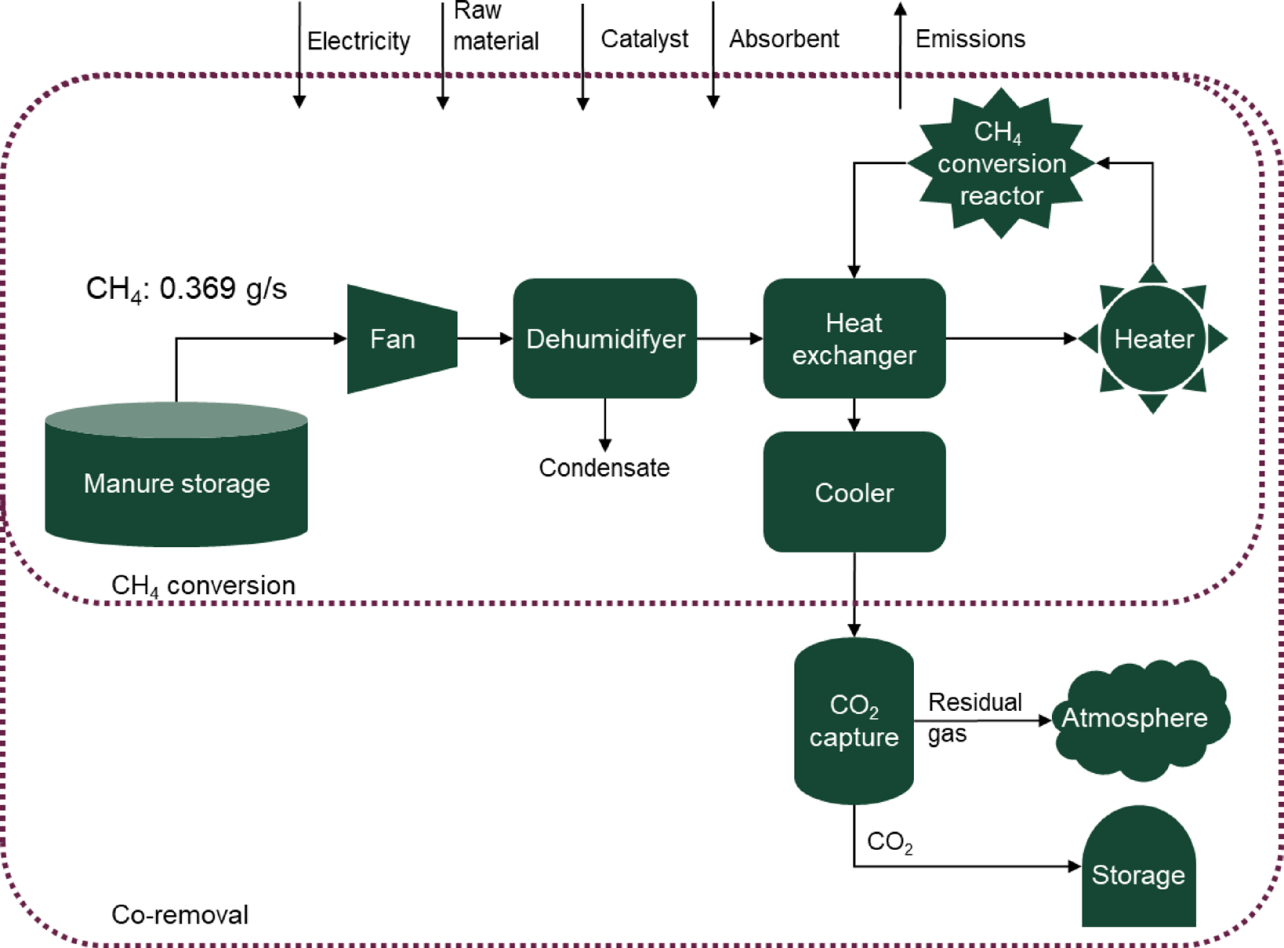
System sketch illustrating the scenarios covered in the study.

#### Scenario A: CH_4_ conversion

In scenario A, the airflow from the covered manure storage headspace was actively ventilated and directed to a catalytic oxidizer where CH_4_ was oxidised to CO_2_ before release into the atmosphere. Different CH_4_ concentrations were achieved by diluting the CH_4_ emissions through varying airflow.

#### Scenario B: co-removal

Once the CH_4_ had been oxidized, following an identical route as in Scenario A, the residual gas flow entered a carbon capture unit to separate the CO_2_ from the rest of the air. Due to the degradation processes in manure forming CO_2_, its concentrations change, to match the amount of CH_4_, creating a 40:60 mix. The CO_2_ flows are described in the SM (Fig. S1 & Table S2). The separated CO_2_ was liquefied and transported to and injected at a geological storage site. According to a previous work on transport and storage for relevant conditions, the captured CO_2_ would require 1.5 GJ/tonne CO_2_ stored and cause 0.10 tonne CO_2_-eq emissions/tonne CO_2_ to be stored^49^.

### Process description and modelling

Modelling of the CH_4_ conversion unit and co-removal via solid sorbent-based adsorption followed the same methodology as presented in Sirigina, et al.^22^, with some modifications made to fit the application of the present study. The chosen CH_4_ concentrations and corresponding gas flows were given as input data (see SM for details). The model output consisted of stream flow rates (Tables S5–S8), energy demand for each process step (Table S11), as well as the capacity and size of the required equipment (Tables S12–S16). The flow sheet for the methane conversion process is shown in Fig. 1 and Fig. S12a in the SM. The outlet stream from the manure storage was directed into the process using a blower. A dehumidifier reduced the inlet moisture content in the stream. The stream was then preheated using the thermal energy of the product stream from the reactor. An electric heater increased the temperature before entering the reactor. The exhaust stream was cooled down after leaving the reactor. For 300 and 1000 ppmv CH_4_, a solid sorbent based vacuum temperature swing adsorption (VTSA)-based process was considered for CO_2_ capture.

For the cases with a 3000 and 10,000 ppmv CH_4_ concentration, a monoethanolamine (MEA) based absorption process was modelled in Aspen Plus and integrated to the CH_4_ conversion unit (Tables S17, S18). Process schematics for co-removal based on adsorption (co-removal) and co-removal based on MEA absorption are shown in the SM (Fig. S12b and c).

The Aspen Process Economic Analyser (APEA) integrated in Aspen Plus was used to estimate the installed weight of the equipment. The weight of all the equipment except the reactor was obtained from APEA. The methodology for sizing the reactor and of heat transfer coefficients (Tables S9 & S10) used in dimensioning the heat exchangers are presented in the SM.

The plant size was inversely related to the CH_4_ concentration, as a low concentration entails a larger volume of air being treated to remove the same total amount of CH_4_. Stainless steel 304 was considered as the default material for all the heat exchangers and the reactor in the CH_4_ conversion unit. Other installations were assumed to consist of 90% steel and 10% concrete as a best estimate. Plant lifetime was set to 20 years.

The catalyst used for CH_4_ oxidation is 6.5% Pd/Al_2_O_3_, and a conversion rate of 95% was considered for the analysis. The catalyst amount required for the conversion was estimated based on the kinetic equation provided in Alyani and Smith^50^. For the conversion rate to be even higher, the catalyst amount would increase exponentially. The right amount of catalyst for each CH_4_ concentration was calculated based on reaction kinetics to match the amount of CH_4_ emitted. The lifetime of the catalyst was set to 10 years, meaning it is exchanged once during the technical lifetime of the plant. The studied catalyst shows reversible inhibition in the presence of water^50^; hence a dehumidifier is needed. It is plausible that a considerable share of ammonia will dissolve in the condensate from the dehumidifier. However, the effects on the process of remaining ammonia are currently unknown and therefore are not considered. Based on the design conditions assumed in the study, other gases, such as N_2_O, were assumed to have passed through the system without undergoing any reaction^51^. We assumed no gas slip occurred within the treatment process since all the components are closed components in the system. An inlet temperature of 330 °C was considered for the catalytic conversion of methane^22,50^. At 300 ppmv, a separate heater was necessary to reach the required reaction temperature, while at the higher CH_4_ concentrations, the excess heat from the oxidised CH_4_ was enough to sustain the reaction. It was necessary to cool the gas stream after the reaction regardless of whether it was exhausted or if CO_2_ was captured.

The choice of CO_2_ capture system was based on energy demand estimated by Kiani et al.^52^ and Sabatino et al.^53^. The chemical absorption system using MEA has higher energy demand at very low concentrations of CO_2_ when compared to the solid sorbent-based capture technology. Therefore, solid sorbent-based technology was considered for the 300 and 1000 ppmv cases, while the MEA based chemical absorption CO_2_ capture technology was considered for 3000 ppmv and 10,000 ppmv cases.

A solid sorbent (APDES-NFC; 3-aminopropylmethyldiethoxysilane - functionalized nanofibrillated cellulose adsorbent) VTSA process was used for the CO_2_ capture at the low CH_4_ concentrations (300 and 1000 ppmv CH_4_). The energy demand for CO_2_ capture through adsorption (excluding the blower) was set to 11.04 GJ/tonne CO_2_^53^. A process based on APDES-NFC was considered due to its similarity with the sorbent used by Climeworks. The case with the maximum productivity was considered and the resulting sorbent requirement was linearly scaled down for the capture of CO_2_, corresponding to the amount in the stream from CH_4_ conversion unit. The regeneration temperature was 110 °C, capture efficiency was 80% and PED was 2.83 GJ/tonne CO_2_ based on Sabatino et al.^53^. Heat integration was not possible at 300 and 1000 ppmv due to the low quality of waste heat stream from the CH_4_ conversion unit. The manufacturing for this case was assumed to equal the DAC plant described by Terlouw, et al.^54^ and scaled linearly to match the capture capacity of our system. For 3000 ppmv and 10,000 ppmv CH_4_ concentrations, a MEA based absorption process was modelled in Aspen Plus and integrated to CH_4_ conversion unit. Heat integration was modelled by using excess heat from the CH_4_ reactor for the regeneration of the absorbent. Process schematics for co-removal based on adsorption (co-removal) and co-removal based on MEA absorption are shown in Fig. S12b and c. The regeneration temperature was ~ 120 °C. The capture efficiency was ~ 89% for the case with 10,000 ppmv CH_4_ concentration, while the capture efficiency was ~ 83% for the case with 3000 ppmv CH_4_ concentration. It was found from the model that approximately 1.5 kg MEA was required as makeup per tonne of CO_2_ capture for the case with 10,000 ppmv CH_4_ concentration, while 1.95 kg MEA was required per tonne of CO_2_ captured for the case with 3000 ppmv of CH_4_ concentration. The amount of MEA required for makeup in our models was similar to the value (1.5 kg) reported in literature^55^. The environmental impact for MEA was obtained from Ecoinvent^38^. The CO_2_ capture plant sizing for the MEA absorbent process was done using the Aspen Process economic analyser in the same way as with other installations.

## Results

The output from the process modelling constituted a part of the LCI and was part of the life cycle PED and CE. Additional results such as specific results from the process model in Aspen can be found in the Supplementary material. As defined in 2.1, a negative net CE denotes lower system emissions than mitigated emissions, whereas a positive net CE is to be interpreted as the system causing more GHG emissions than were being mitigated per functional unit.

### Scenario A: CH_4_ conversion

**Fig. 2:**
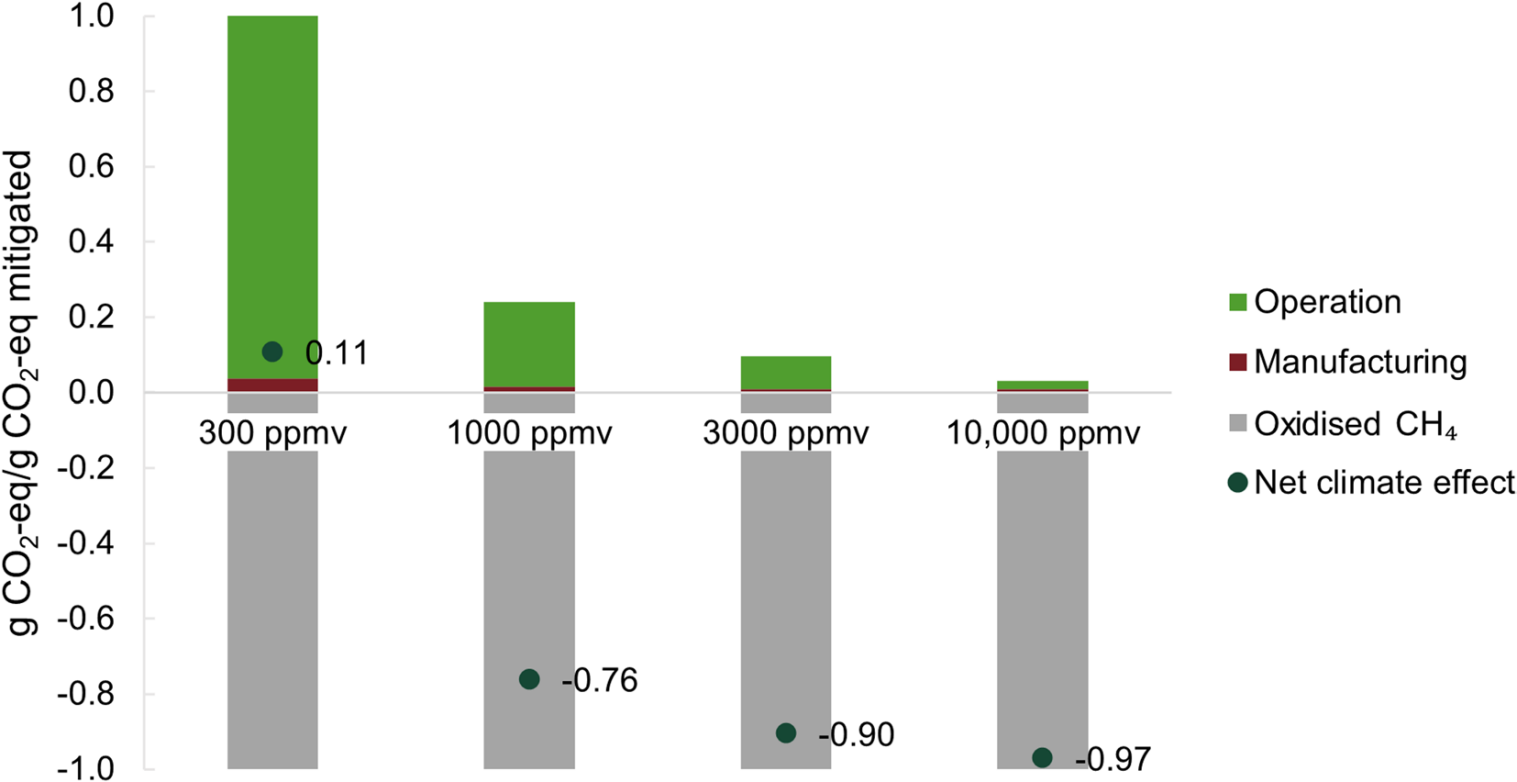
The climate effect of processes necessary for oxidising CH_4_ as well as the net effect. The study is designed to remove an equivalent amount of CH_4_ in each scenario but at different concentrations, hence the grey bar has the same value for all four studied scenarios.

The CE for oxidising CH_4_ at the four different concentrations is shown in Fig. 2, displaying the positive and negative climate contribution as well as the net value represented by the difference between the two. The CE was clearly non-linear with an improving net-effect with increasing CH_4_ concentrations. A total amount of 0.37 g CH_4_/s (10.3 g CO_2_-eq) was oxidised, and 0.019 g CH_4_/s (0.54 g CO_2_-eq) exited with the waste gas, corresponding to the 95% conversion capacity of the catalyst. In accordance with the definition of the functional unit, this oxidised amount equals − 1.0 g CO_2_-eq.

The additional GHG emissions were divided into emissions originating from the manufacturing and those from the operations phase. The results for the end-of-life phase were too low to be clearly displayed in any graphic and were therefore included in the manufacturing phase. The emissions from the operations phase made the predominant contribution (98%, 96%, 93% and 83% at a 300, 1000, 3000 and 10,000 ppmv CH_4_ concentration, respectively). However, as the absolute additional emissions decreased for higher CH_4_ concentrations, the relative impact of the plant manufacturing increased, corresponding to 2%, 4%, 7% and 17%, respectively (Fig. S2 in the SM). The GHG emissions from the operation consist of direct emissions from the process, and indirect emissions from the energy demand. The direct emissions are what is left in the exhaust. These are counted as climate neutral due to their biological origin and the fact that in the reference case, they would all have reached the atmosphere. However, they impact the results via the functional unit, which is made up by the mitigated emissions. The indirect emissions depend on the energy source used to power the process.

The total PED was 31, 6.3, 2.3 and 0.77 kJ/g CO_2_-eq mitigated from the low to high CH_4_ concentrations, respectively. The energy required for operation made up a significant share of the total PED (Fig. S2 in SM). In absolute numbers, it decreased with increasing CH_4_ concentration. Lower concentrations of CH_4_ led to larger volumes of air being treated to mitigate a given quantity of CO_2_-eq, hence the PED for pressure loss, dehumidifying and heating the air increase. At all four CH_4_ concentrations, the blower constitutes the largest share of the PED for operation, followed by the dehumidifier (Fig. S3 in SM).

The manufacturing of the dehumidifier was associated with high PED and CE. In the 10,000 ppmv case, this amounted to almost 60% of the PED and 50% of the CE (Fig. S5 in SM). The palladium for the catalyst used to initiate the CH_4_ oxidation asserted a high climate impact, and at the lowest concentration, which requires the largest catalyst amount, this constituted almost 65% of the overall climate effect from manufacturing. The recuperator was also demanding to manufacture (Fig. S5 in SM). However, as CH_4_ concentrations increased so does the heat discharge from the oxidation process, meaning the size of the recuperator could be significantly decreased. At the lowest concentrations, additional heat was required to reach a high enough temperature for oxidation to initiate, but at 1000 ppmv CH_4_ and above, the heat transfer in the recuperator is sufficient to sustain the reaction.

### Scenario B: co-removal

From a life cycle perspective, climate efficiency was worsened when CCS was added (Table 1). This is mainly due to the much higher GWP of CH_4_ but also due to the additional process steps required for CCS, which slightly increase the PED and cause additional GHG emissions (Figs. S4 & S8 in SM). When summarizing the effects of the oxidized CH_4_ with the captured CO_2_, the mitigation equals 12.9, 11.7, 11.5 and 11.4 g CO_2_-eq/s from the lowest to highest CH_4_ concentrations, respectively. In accordance with the definition of the functional unit, this oxidised amount equals − 1.0 g CO_2_-eq.

**Table 1:** Changes in climate effect (g CO_2_-eq emitted/g CO_2_-eq removed) and PED for co-removal compared to CH_4_ conversion.

CH_4_ concentration (ppmv)	300	1000	3000	10,000
Net climate effect (CH_4_ conversion)	+ 0.11	-0.76	-0.90	-0.97
Manufacturing (CCS)	+ 0.0057	+ 0.013	+ 0.0083	+ 0.016
Operation (CCS)	+ 0.011	+ 0.13	+ 0.057	+ 0.00057
Transport and storage (CCS)	+ 0.015	+ 0.011	+ 0.0097	+ 0.0092
Stored CO_2_	**-0.21**	**-0.15**	**-0.13**	**-0.13**
Net climate effect (co-removal)	**+ 0.14**	**-0.62**	**-0.83**	**-0.94**
Change in GWP (absolute)	**+ 0.073**	**+ 0.18**	**+ 0.15**	**+ 0.10**
Change in GWP (relative)	**+ 65%**	**+ 24%**	**+ 17%**	**+ 11%**
Change in PED (relative)	**+ 2%**	**+ 51%**	**+ 82%**	**+ 59%**

The PED increased due to the added capture unit, mainly due to the energy required for the regeneration of the sorbent. The energy demand from operating CO_2_ capture alone was higher than CH_4_ conversion in the cases with medium and high CH_4_ concentrations.

**Fig. 3:**
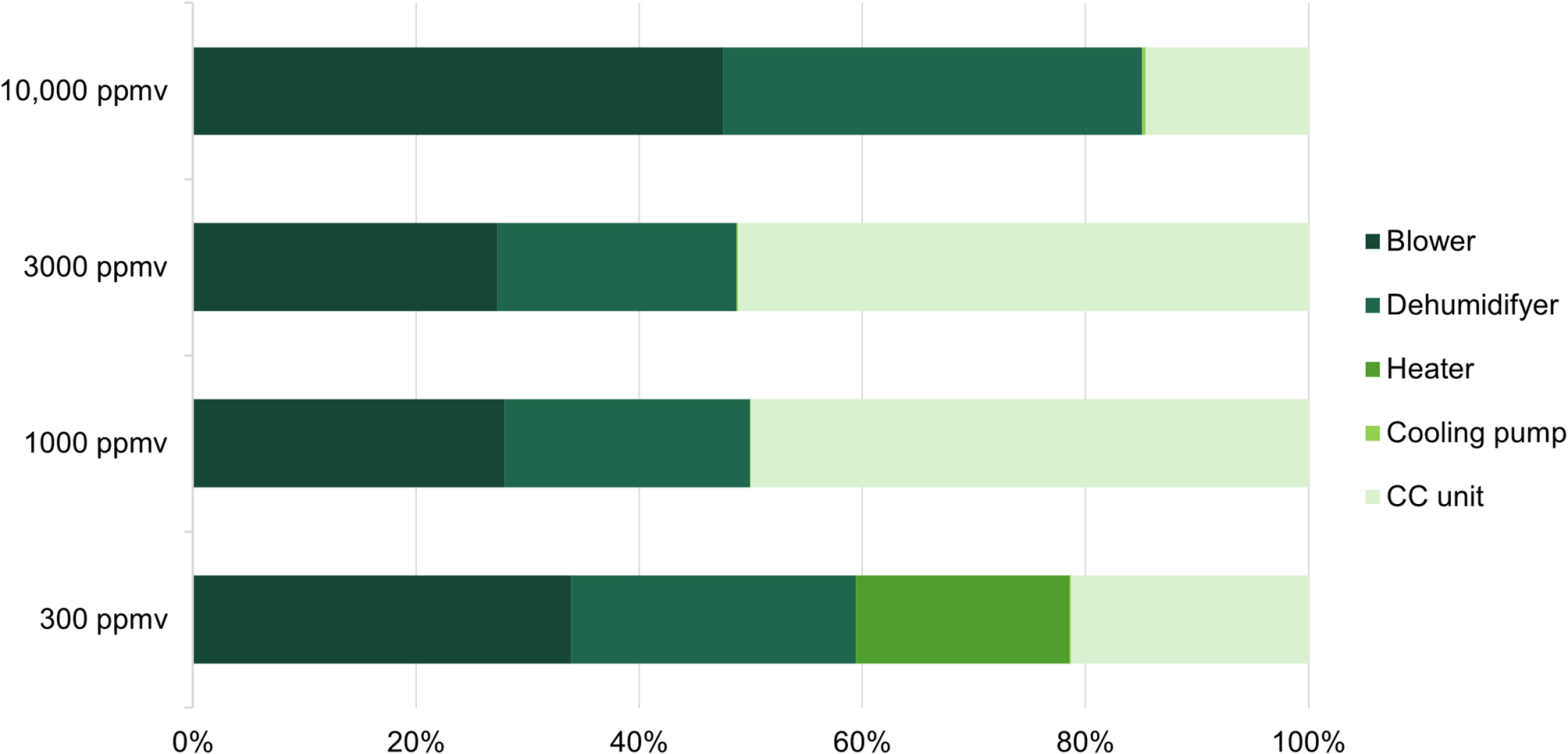
The share of primary energy demand when operating the CH_4_ oxidation and CO_2_ capture processes at the four studied CH_4_ concentrations, divided between the energy using components.

The CO_2_ capture process required additional energy use, which was especially visible for the medium concentrations (Fig. 3). The PED for the CO_2_ capture process was highly dependent of the concentration of both CH_4_ and CO_2_. At the highest CH_4_ concentration, the energy generated from the CH_4_ oxidation reaction could be utilized in the amine regeneration, lowering the need for additional heat.

The manufacturing of the CO_2_ capture components added a considerable contribution to the overall manufacturing emissions, around 40% for all three cases (Fig. S6 in SM).

### Sensitivity analysis

The following results are for the sensitivity analyses performed for the CH_4_ conversion scenario. Results for sensitivity analyses for the co-removal scenario can be found in the SM (Figs. S9 and S11).

#### Energy source

**Fig. 4:**
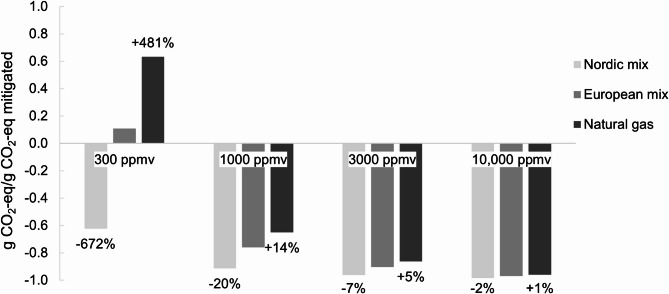
Sensitivity analysis for the impact of emissions factor for electricity on the net climate effect for CH_4_ conversion at the four studied CH_4_ concentrations. The data labels show the relative increase/decrease compared to the main scenario (European mix).

In a sensitivity analysis, the impact of using different emission factors and PEFs for electricity was evaluated. The European mix used in the main scenario was exchanged for natural gas power and a Nordic consumption mix. A comparison shows this had a determining effect on the net CE for the low CH_4_ concentration (Fig. 4), while the impact is more modest for the higher CH_4_ concentration.

The primary energy factor differs between the different electricity sources, which affects the PED. For the natural gas, this leads to a PED decrease of between 8.0 and 11%; for the Nordic mix, the PED decrease was between 23 and 25%.

#### Climate metrics

**Fig. 5:**
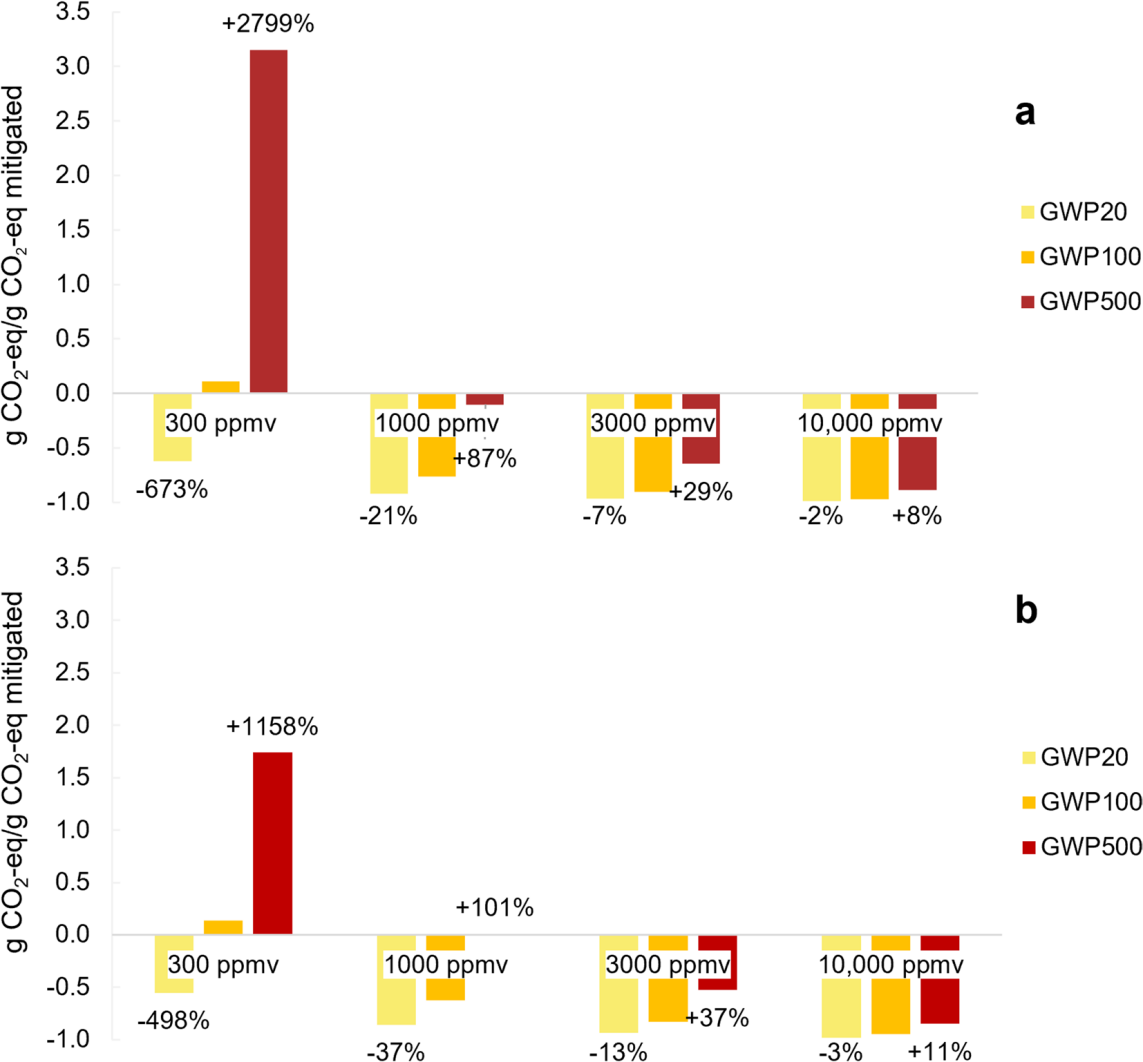
Sensitivity analysis highlighting the choice of metric on the net climate effect for CH_4_ oxidation at the four modelled CH_4_ concentrations. The data labels show the relative increase/decrease compared to the main scenario (GWP100). CH_4_ conversion (**a**) and co-removal (**b**).

A sensitivity analysis for the impact of the choice of climate metric was evaluated, highlighting GWP20 and GWP500 in comparison to the main case which uses GWP100 (Fig. 5). The shorter the chosen time horizon for the metric, the better the system performance appears. At the low CH_4_ concentration, the system net CE varies greatly depending on the metric used. For the medium and high CH_4_ concentrations, the impact is visible but more modest.

**Fig. 6:**
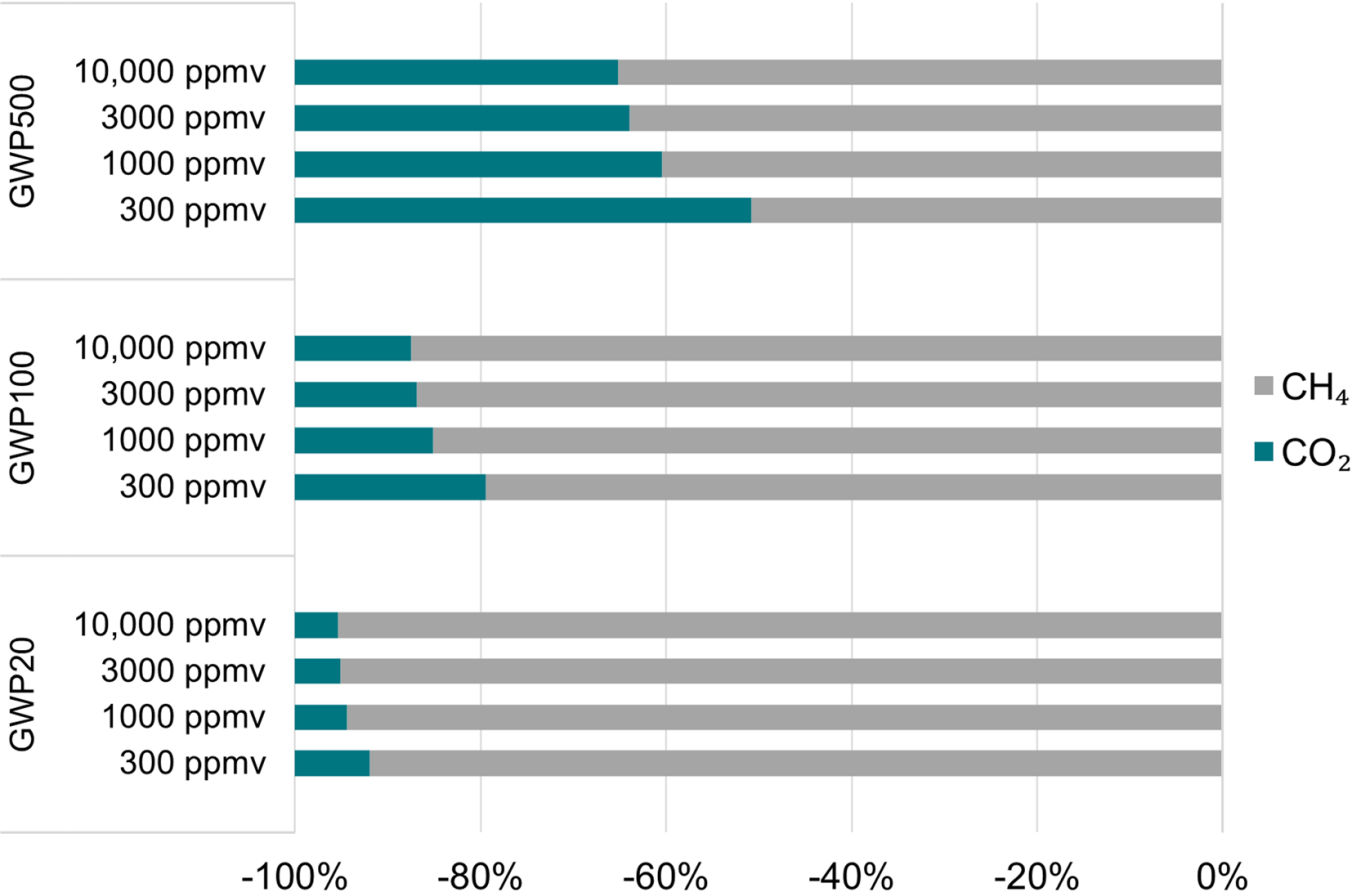
The achieved climate benefit of the co-removal is shared by CO_2_ and CH_4_. The figure shows the relative contribution of each gas at the four CH_4_ concentrations modelled using different climate metrics.

The GWP reduction for the process was shared by CH_4_ and CO_2_. With the use of the different metric time horizons, the relative contribution of each gas varied (Fig. 6).

The climate metric affects the functional unit, which in turn affects the results for PED. In these two cases, the change would be a 66% decrease in PED per g CO_2_-eq mitigated for GWP20, and a 275% increase for GWP500.

Through a combined analysis of electricity emissions factor and climate metric, an interval of the CE of the technology under different conditions and assumptions can be identified (Figs. S10 and S11).

## Discussion

The results confirmed that the CH_4_ concentration was the most influential factor for the net CE of the system. It was inversely proportional to the energy demand for operation due to the large volumes of air being treated per gain in reduced climate impact at low concentrations of CH_4_ (i.e. the relation between the number of molecules of H_2_O to remove and N_2_ molecules to heat compared to CH_4_ oxidised). The net CE was positive (Fig. 2) at the lowest CH_4_ concentration modelled in this study (300 ppmv), i.e. the CH_4_ conversion process contributed to global warming from a life cycle perspective much due to the GHG emissions from operation, outweighing the gain from the oxidised CH_4_. However, a sensitivity analysis highlighted the importance of increasing the share of renewable energy sources, as switching to a renewable electricity mix (modelled as the current consumption mix of the Nordic countries) improved the net GWP more than 6 times compared to the European mix (Fig. 4). This resulted in a net negative CE also for the lowest CH_4_ concentration. The impact of the electricity mix was especially clear at the low CH_4_ concentration due to its high energy demand. For CH_4_ concentrations from 1000 to 10,000 ppmv, a net-negative CE could be reached even with natural gas-powered electricity, although at worsened net CE. A higher share of renewables also lowered the PED as these energy sources have lower primary energy factors than fossil energy sources.

To evaluate and compare the climate impact of emitting or mitigating GHGs, a conversion metric must be chosen. The GWP100 is the most widespread metric used in both policy and science, although it has long been criticised for not fairly representing the actual temperature response, especially underestimating short term effects caused by, for example, CH_4_ e.g. in^6,56,57^. The sensitivity analysis of the climate metric (Fig. 5) illustrates how a shorter time horizon weights the impact of CH_4_ mitigation more than a longer time horizon. GWP20 emphasises the impact of mitigating the short-term effects of CH_4_, resulting in net-negative CE for all three concentrations, both with and without CO_2_ capture. GWP500 instead put more emphasis on the long-term impact of CO_2_, giving a clear lowering of the net CE compared to the GWP100 results.

Due to the strong warming effect of CH_4_ compared to CO_2_, no matter the metric used in this study, avoiding the impact of CH_4_ played a decisive role compared to the benefits of also capturing the subsequent CO_2_, ranging from around 50% of the total climate benefit attributed to CH_4_ and 50% from CO_2_ for GWP500 to 92% for CH_4_ and 8% for CO_2_ for GWP20 (Fig. 6). No matter the concentration and metric, the net CE decreased when CO_2_ capture was introduced due to the additional energy (Table 1) and material demand compared to sole CH_4_ oxidation. However, depending on the studied timescale, the prioritisation between CH_4_ and CO_2_ could differ, as targeting CH_4_ emissions could deliver significant short-term effects. From a longer time perspective, however, the accumulation of CO_2_ in the atmosphere is what will largely determine the level the mean global temperature will reach and remain at due to its longer residence time^58^.

The study identified the blower, the dehumidifier and the catalyst demand as important hotspots for both energy demand and CE (Fig. 3). Since the process energy had such a major impact on the overall result, especially at low concentrations, technological improvements could have a substantial effect on overall performance and feasibility. Energy demand for the blower can be reduced by minimizing pressure drops throughout the process. The development of a less water-inhibited catalyst material with a lower carbon footprint would improve the system CE directly at the manufacturing stage (Figs. S6 & S7 in SM) and indirectly by reducing or eliminating the need for the energy demanding dehumidification step. Palladium minerals are rare and have wide uses, from catalysts to jewellery, electronics and fuel cells. Hence, like with other metals important for the green transition, there are risks such as rising prices and tightened supply in the future^59^. Furthermore, a lower minimum temperature difference in the recuperator could reduce the energy demand, although this would increase the heat transfer area of the recuperator. As part of the PED for operation consists of thermal energy, it may be possible to connect a secondary renewable energy source or waste heat to improve the net CE. All these aspects are especially pronounced at low CH_4_ concentrations due to the high energy and raw material demand per CO_2_-eq mitigated.

For manure storage, the CH_4_ emissions at each moment in time vary based on a number of factors. As this is expected to impact the operation of the machinery as well as system efficiency and subsequent energy use, we chose to conduct this study as a snapshot of a moment in time with static conditions, with the four studied concentrations covering a large interval of conditions. This approach was an attempt to decrease complexity and be able to perform initial evaluation and information gathering on the most important parameters affecting strategies for design and operation of this novel system. Under practical operating conditions, manure level, outdoor temperature and energy source would have a decisive impact on the net CE. The rate of CH_4_ emitted is closely linked to the storage temperature, and the relatively large energy demand for operation constituted a noticeable impact on the net CE. Therefore, one could expect that this type of system would deliver the largest climate benefits if implemented in parts of the world with a warm climate and a substantial renewable electricity supply, and on farms with large herds of livestock and effective manure collection systems. The larger the share of manure collected, the greater the climate benefit that could be achieved for the investment per animal. It is also vital that the entire value chain required for CH_4_ oxidation and CO_2_ capture and storage can be carried out with a minimum of GHG slip and performed in a climate friendly and energy efficient manner to reach the highest possible total climate benefit and maintain high credibility for the GGR concept.

This study evaluated the technology from the perspectives of PED and CE. However, other types of environmental impacts may also be relevant. The MEA used for CO₂ capture is toxic and acidic, posing risks to organisms and the local environment^60^. CO₂ capture and exhaust cooling also require water, which can increase freshwater consumption. On the positive side, the technology may reduce NH₃ emissions, as part of the ammonia dissolves in the condensate from the dehumidifier. This may help mitigate e.g. acidification, aerosol formation and odour. Indirect environmental effects may also occur elsewhere or over time — for example, from aluminium and palladium mining, which can involve hazardous chemicals, affect human health, and contribute to land use change and biodiversity loss. These potential impacts warrant further investigation and quantification in future studies.

Considering the promising net CE presented in this study, it seems reasonable to continue exploring technologies for the conversion of CH_4_ with and without subsequent CO_2_ capture to analyse its prospects for implementation under different conditions, such as scale, location and CH_4_ concentration. Some relevant research topics for future studies could be investigating practical constraints on the equipment and materials, techno-economic analysis, and experimentation to confirm modelling results. There are also options to increase system efficiency and increased systems integration through, for example, carbon capture and utilisation. In the future, more detailed case studies that examine feasibility under certain conditions with more case specific and time-dynamic data for a particular application should be used, whether it be manure storage or some other unabated CH_4_ emissions source.

The low CH_4_ concentrations analysed in this study could allow for the targeting of emission sources where GGR has not been considered previously, such as the agrifood sector, which accounts for over half of overall CH_4_ emissions. There is currently no technology to completely eliminate non-CO_2_ GHG emissions from agriculture, and improvements in carbon efficiency and emission reduction measures risk being partly counteracted by the increased primary production demand necessary for feeding an increasing world population^61^. The burden on reduced GHG emissions in other sectors would increase unless unabated agricultural emissions could be sequestered or compensated for through additional GGR. Further research on ways to reduce global warming via CH_4_ could improve the technology used and identify the most promising areas of implementation to maximise efficiency.

## Conclusions

This study presents a thermal catalytic technology capable of delivering climate mitigation by oxidising low-concentration CH_4_ emissions, below the point of ignition, with and without subsequent CO_2_ capture and storage. The results showed that the PED was highly dependent on the CH_4_ concentration. At 300 ppmv CH_4_, the process yielded a slightly positive net climate effect, but achieved a net-negative climate effect at CH_4_ concentrations of 1000 ppmv and above. The prominent impact of the process energy demand rendered the energy source decisive. When the assumed European electricity mix was replaced with a lower-emission Nordic mix, a net-negative climate effect was reached at all four studied CH_4_ concentrations. Changing the climate metrics from GWP100 to GWP20 also led to net-negative results across all concentrations, as it emphasises the short-term warming impact of CH₄. At CH_4_ concentrations above 3000 ppmv, a lower energy demand in combination with the increasing feasibility of utilising excess heat from the CH_4_ conversion process enabled net-negative effects even with natural gas operation, and regardless of the climate metric. Nonetheless, using a low emissions energy and a high energy efficiency is key to maximise the benefit and credibility of the technology avoid wasting limited renewable energy resources on GGR.

Adding CO₂ co-removal and storage worsened the net climate effect at all concentrations due to increased PED and the dominant short-term impact of CH₄ conversion. Targeting the most concentrated CH_4_ emission sources possible is thus the most energy efficient approach. However, system improvements- such as reducing pressure drops in the blower and dehumidifier and developing more durable, low-footprint catalysts- could further increase energy efficiency. Such developments would increase the system efficiency to reach net-negative climate effect at even lower CH_4_ concentrations, thus allowing catalytic conversion of CH_4_ to target and efficiently abate a wider range of CH_4_ emission sources.

## Supplementary Information

Below is the link to the electronic supplementary material.

Supplementary Material 1

## Data Availability

Supplementary material associated with this article can be found in the online version.

## Abbreviations

CE: Climate effect
CCS: Carbon capture and storage
CDR: Carbon dioxide removal
CH_4_: Methane
CF: Carbon footprint
CO_2_: Carbon dioxide
ED: Energy demand
GGR: Greenhouse gas removal
GGRT: Greenhouse gas removal technology
GHG: Greenhouse gas
GWP: Global warming potential
kJ: Kilo Joule (10^3^ joule)
LCA: Life cycle assessment
MJ: Mega Joule (10^6^ joule)
PED: Primary energy demand
