# Supplementary material for: Reduced life cycle climate impact from manure through catalytic methane conversion and carbon dioxide removal
Source: Sci Rep. 2025 Dec 10;15:43580. doi: 10.1038/s41598-025-27609-2 (PMC12698671; doi:10.1038/s41598-025-27609-2)
Supplement: Supplementary file 1 — Supplementary Material 1 [file 41598_2025_27609_MOESM1_ESM.docx]

**Reduced life cycle climate impact from manure through catalytic methane conversion and carbon dioxide removal**

**Supplementary material**

Emma Bromark^a,c^ *, Devesh Sathya Sri Sairam Sirigina^b,d^, Shareq Mohd Nazir^b,e^, Pernilla Tidåker^a,f^_,_ Åke Nordberg^a,g^ , Per-Anders Hansson^a^

Submitted: 2025-03-28

^a^ Department of Energy and Technology, Swedish University of Agricultural Sciences, Lennart Hjelms väg 9, 756 51 Uppsala, Sweden

^b^ Department of Chemical Engineering, KTH Royal Institute of Technology, Teknikringen 42, 11428 Stockholm, Sweden

^c^ https://orcid.org/0000-0003-3135-8315

^d^ https://orcid.org/0000-0002-4702-8081

^e^ https://orcid.org/0000-0002-4056-0454

^f^ https://orcid.org/0000-0002-4108-4680

^g^ https://orcid.org/0000-0002-1662-9730

* Corresponding author

E-mail addresses: [emma.bromark@slu.se](mailto:emma.bromark@slu.se) , [sirigina@kth.se](mailto:sirigina@kth.se) , [smnazir@kth.se](mailto:smnazir@kth.se) , [pernilla.tidaker@slu.se](mailto:pernilla.tidaker@slu.se) , [ake.nordberg@slu.se](mailto:ake.nordberg@slu.se) , [per-anders.hansson@slu.se](mailto:per-anders.hansson@slu.se)

Table of contents

[Part A 3](#_Toc212034910)

[Background 3](#_Toc212034911)

[Life cycle assessment 4](#_Toc212034912)

[Additional results from the study 5](#_Toc212034913)

[Manufacturing 8](#_Toc212034914)

[Net climate impact 10](#_Toc212034915)

[Sensitivity analysis 11](#_Toc212034916)

[Best & worst case 11](#_Toc212034917)

[Part B: Process modelling 13](#_Toc212034918)

[Stream data 13](#_Toc212034919)

[Aspen output: Energy and material demand for the treatment process 15](#_Toc212034920)

[Dimensioning of plant 16](#_Toc212034921)

[Dimensioning of the reactor 19](#_Toc212034922)

[CO_2_ Capture by absorption 19](#_Toc212034923)

[References 22](#_Toc212034924)

# Part A

## Background

Methane is prevalent at a variety of concentrations. Due to the biological origin of CH_4_ emissions from food systems, it is often emitted over large unconfined areas and is quickly diluted in the atmosphere. The emission rate of biological sources may vary due to conditions such as moisture and temperature. In sum, the emissions are great and pose a noticeable impact on the climate. However, due to these characteristics, they may be hard to address (Abernethy et al, 2023). Hence, it is hard to give a robust answer to how high CH_4_ concentrations are at different emission sources, but to give some form of reference, we present conditions at some different emission sources in Table S1.

Table S1. Some CH_4_ concentrations for reference. Many measurements of CH_4_ emissions focus on the amounts/volume flow of CH_4_ and do not report concentration as the CH_4_ disperse quickly in the atmosphere.

| Emission source | CH_4_ concentration | References |
| --- | --- | --- |
| Coal mine ventilation | 0.1-1%v | Ursueguia et al., 2021 |
| Liquid manure storage | 760-8400 ppmv | Melse & van der Werf, 2005 |
| Naturally ventilated stable | 15-152 ppmv | Tabase et al., 2023 |
| Biogas | 40-75%mol | Poblete et al., 2020 |
| Diluted landfill gas | 7 % | Fjelsted et al., 2020 |
| Natural gas infrastructure | 100-50,000 ppm | Jia et al., 2025 |
| Rice field | 7 ppm | Pazhanivelan et al., 2024 |
| Digestate | 200-900 ppm | Gålfalk et al., 2024 |
| Atmosphere | 1.9 ppm | NOAA, 2024 |

Figure S1 and Table S2 describe the assumptions behind the thermal catalytic treatment process and resulting CO_2_ flows.


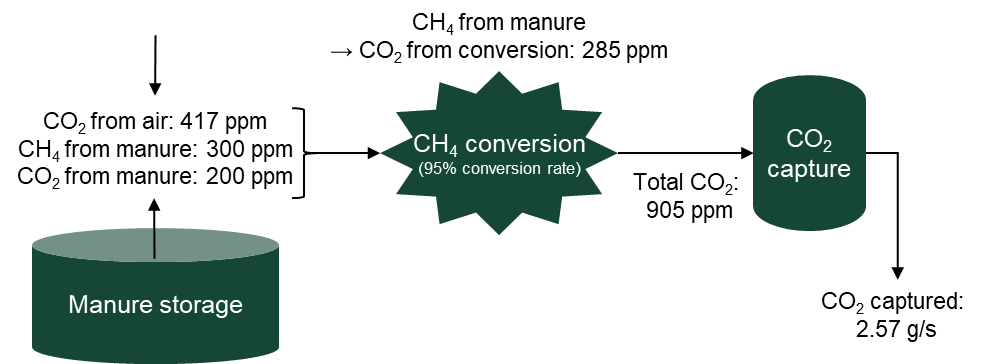


Figure S1. Schematic illustration of CO_2_ flows with data for the 300 ppmv scenario presented in the figure.

Table S2. Concentrations of GHG (ppmv) at the four studied CH_4_ concentrations.

| CH_4_ concentration | 300 | 1000 | 3000 | 10 000 |
| --- | --- | --- | --- | --- |
| CO_2_ from air | 417 | 417 | 417 | 417 |
| CO_2_ from manure | 200 | 667 | 2000 | 6667 |
| CO_2_ from oxidation | 285 | 950 | 2850 | 9500 |
| CO_2_ entering capture unit | 905 | 2034 | 5267 | 16 584 |
| CO_2_ captured (g/s) | 2.57 | 1.74 | 1.50 | 1.42 |

## Life cycle assessment

This section presents data and assumptions used in the life cycle assessment in Table S3 and Table S4.

Table S3. Core data used for the life cycle assessment.

|  | Value | Data source |
| --- | --- | --- |
| Electricity sources  European mix  Nordic mix  Natural gas | 293  93  436 | Bastos (2020)  Sandgren and Nilsson (2021)  Quaschning (2022) |
| Primary energy factors  European mix  Nordic mix  Natural gas | 2.3  1.7  2.0 | Cogen Europe (2017)  Sweco (2021)  Calculated based on a 50% efficiency |
| Climate metrics  GWP20  GWP100  GWP500 | 79.7  27  7.2 | IPCC (2021)  Assessment report 6  Table 7.15 |

Table S4. Overview of assumptions in the life cycle assessment modelling.

|  | Data | Comment |
| --- | --- | --- |
| Raw materials  Steel  Palladium  Aluminium  Concrete  Monoethanolamine | Ecoinvent  V 3.9.1 | Market activities (including both raw material extraction and transport) were used.  End of life was represented by waste activities for each raw material.  IPCC 2021 cutoff was used for the GWP sensitivity analysis. |
| Technical lifetime | 25 years |  |
| Catalyst | 6.5% Pd/Al_2_O_3_ | For CH_4_ conversion |
| Catalyst lifetime | 10 years | Exchanged twice during plant lifetime |
| CH_4_ conversion rate | 95% |  |
| CH_4_ conversion conditions | 330 °C | Adiabatic conditions |
| CO_2_ capture technology | APDES-NFC  MEA | 300 and 1000 ppmv  3000 and 10,000 ppmv |
| CO_2_ capture efficiency | 80%  83%  89% | 300 and 1000 ppmv  3000 ppmv  10,000 ppmv |
| Solid sorbets capture facility | Terlouw *et al.* (2021) | Scaled linearly to fit this case |
| Manure emissions | 0.369 g CH_4_/s | Manure from high productivity dairy cattle in a cool, temperate, moist climate (Table 10.14) (IPCC 2019) |

## Additional results from the study

This section presents some additional results to the main text through the following figures:

*Figure S2. The share of primary energy demand for the treatment of GHG emissions from manure storage at the four studied CH4 concentrations, divided between manufacturing (mainly raw material extraction and processing) and operation. Emissions related to end of life are included in the manufacturing as they were too small to be clearly displayed in the figure.*

*Figure S3. The share of primary energy demand for running the CH4 oxidation process at the four studied CH4 concentrations. Divided over the energy using components used in the process. The contribution of the cooling pump is too small to view in the figure.*

*Figure S4. The share of the additional emissions causing the positive contribution to the system climate effect at the four studied CH4 concentrations for the co-removal scenario. The GHG emissions from the system can be attributed to either the manufacturing step (mainly raw material extraction and processing), the operations phase (energy related emissions) or managing the captured CO2.*

*Figure S5. Primary energy demand for producing the necessary components for the CH4 conversion process.*

*Figure S6. The share of the climate effect from producing necessary components for the CH4 conversion process.*

*Figure S7. The share of the climate effect from producing necessary components for the co-removal process. The absolute values for the CH4 conversion are identical to Figure S6.*

*Figure S8. Net climate effect for the co-removal process with subsequent CO2 capture at the four studied CH4 concentrations.*

*Figure S9. Sensitivity analysis of the impact of the emissions factor for electricity on the net climate effect for co-removal at the four studied CH4 concentrations. The data labels show the relative increase/decrease compared to the main scenario (European mix).*

*Figure S10. Net climate effect of CH4 conversion for the four modelled CH4 concentrations. The data labels show the relative increase/decrease compared to the main scenario (GWP100 and European mix).*

*Figure S11. Net climate effect of co-removal for the four modelled CH4 concentrations. The data labels show the relative increase/decrease compared to the main scenario (GWP100 and European mix).*


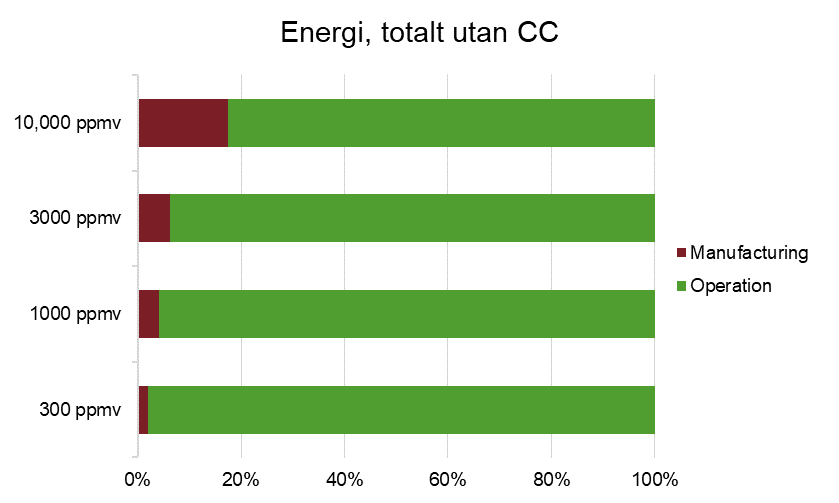


Figure S2. The share of primary energy demand for the treatment of GHG emissions from manure storage at the four studied CH_4_ concentrations, divided between manufacturing (mainly raw material extraction and processing) and operation. Emissions related to end of life are included in the manufacturing as they were too small to be clearly displayed in the figure.


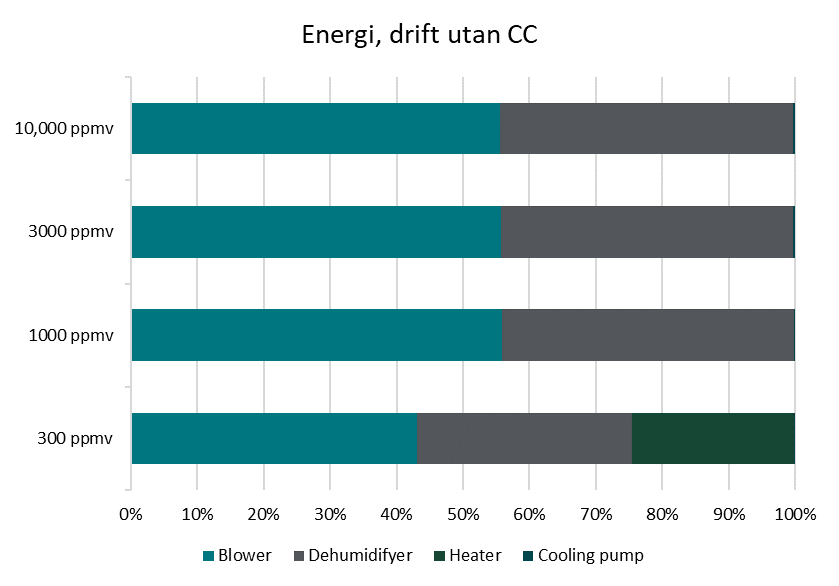


Figure S3. The share of primary energy demand for running the CH_4_ oxidation process at the four studied CH_4_ concentrations. Divided over the energy using components used in the process. The contribution of the cooling pump is too small to view in the figure.


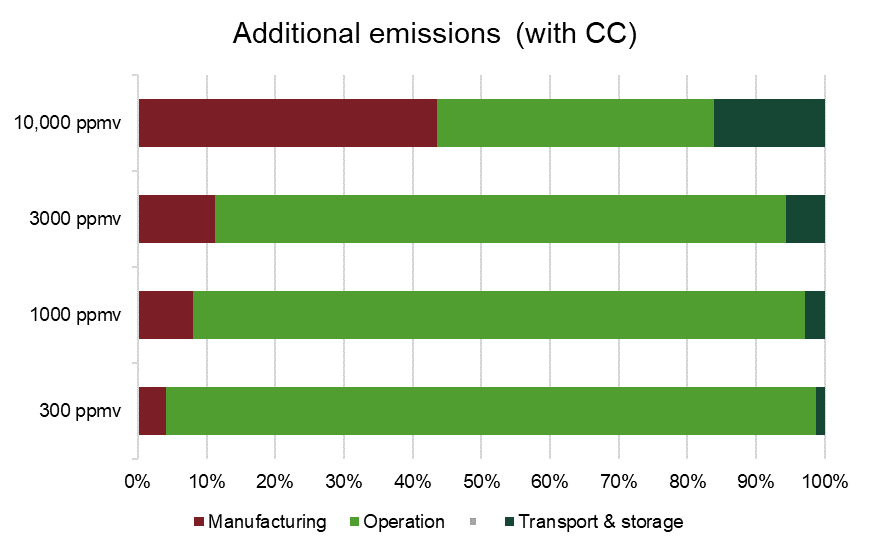


Figure S4. The share of the additional emissions causing the positive contribution to the system climate effect at the four studied CH_4_ concentrations for the co-removal scenario. The GHG emissions from the system can be attributed to either the manufacturing step (mainly raw material extraction and processing), the operations phase (energy related emissions) or managing the captured CO_2_.

### Manufacturing

At the lowest concentrations, additional heat was required to reach a high enough temperature for oxidation to initiate, but at 1000 ppmv CH_4_ and above, the heat transfer in the recuperator is sufficient to sustain the reaction.


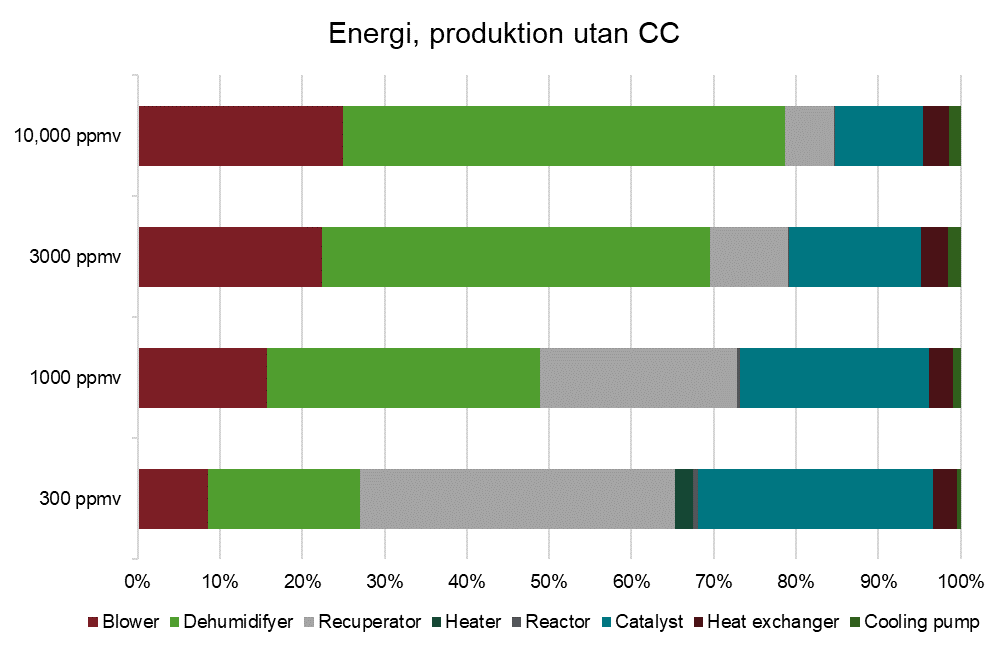


Figure S5. Primary energy demand for producing the necessary components for the CH_4_ conversion process.


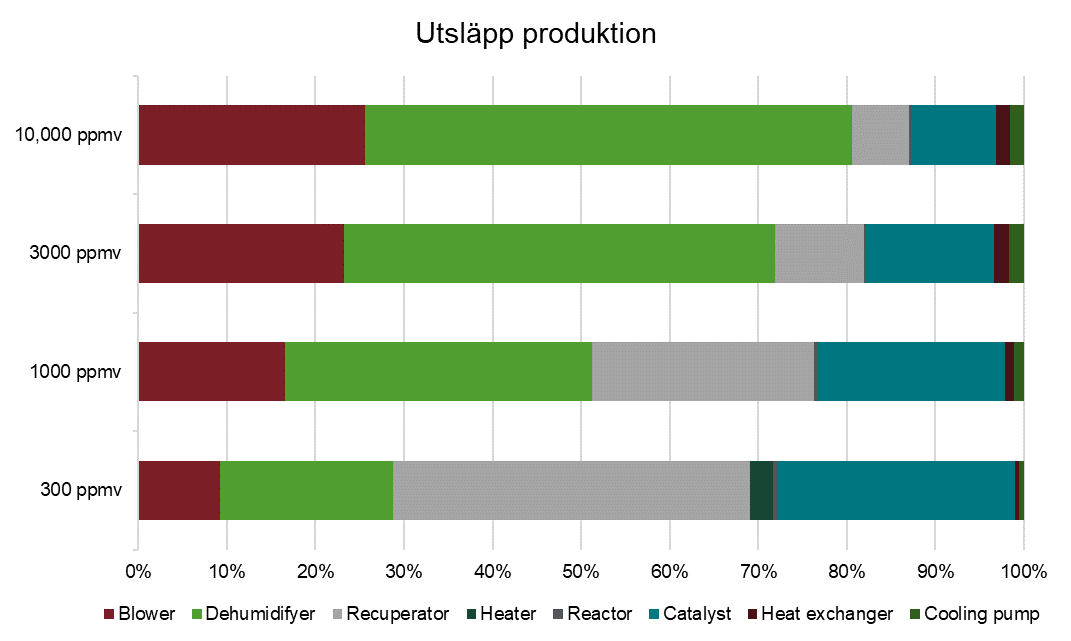


Figure S6. The share of the climate effect from producing necessary components for the CH_4_ conversion process.

The impact of the carbon capture unit is largely due to the sorbent (Figure S7).


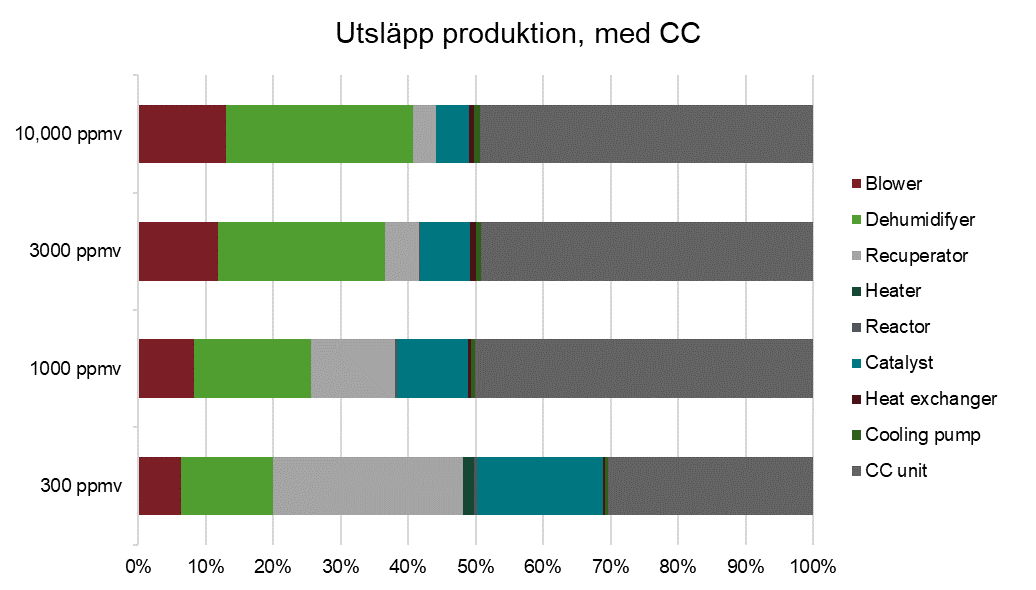


Figure S7. The share of the climate effect from producing necessary components for the co-removal process. The absolute values for the CH_4_ conversion are identical to Figure S6.

### Net climate impact


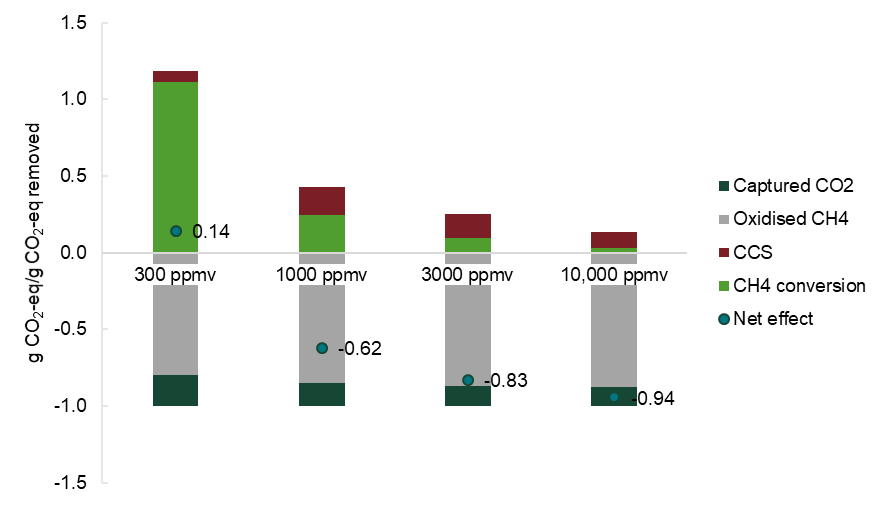


Figure S8. Net climate effect for the co-removal process with subsequent CO_2_ capture at the four studied CH_4_ concentrations.

### Sensitivity analysis

This section presents the same sensitivity analysis as was presented in the article text but for the co-removal scenario.


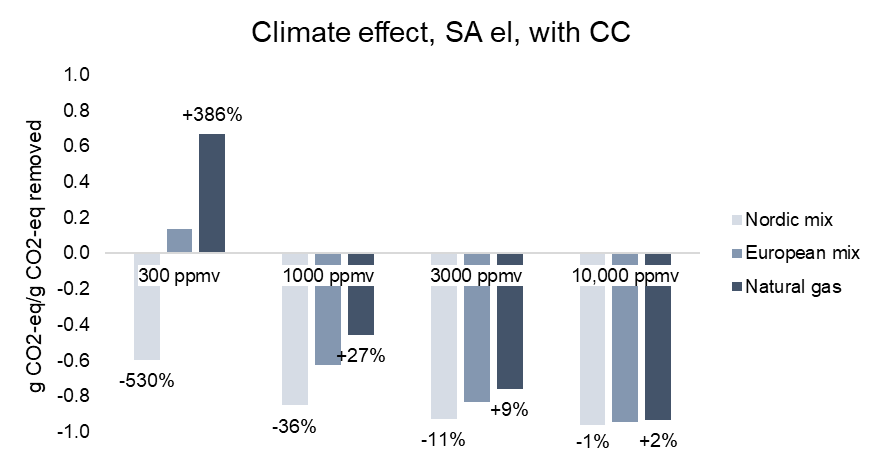


Figure S9. Sensitivity analysis of the impact of the emissions factor for electricity on the net climate effect for co-removal at the four studied CH_4_ concentrations. The data labels show the relative increase/decrease compared to the main scenario (European mix).

### Best & worst case

To show the full range of possible results, we put together scenarios which give the maximum and minimum net climate effect. “Best” and “worst” refers to the quantitative value and does not reflect any qualitative valuation of the authors.

The “best case” scenario is a combination of GWP20 and Nordic electricity mix.

The “worst case” scenario is a combination of GWP500 and natural gas power.


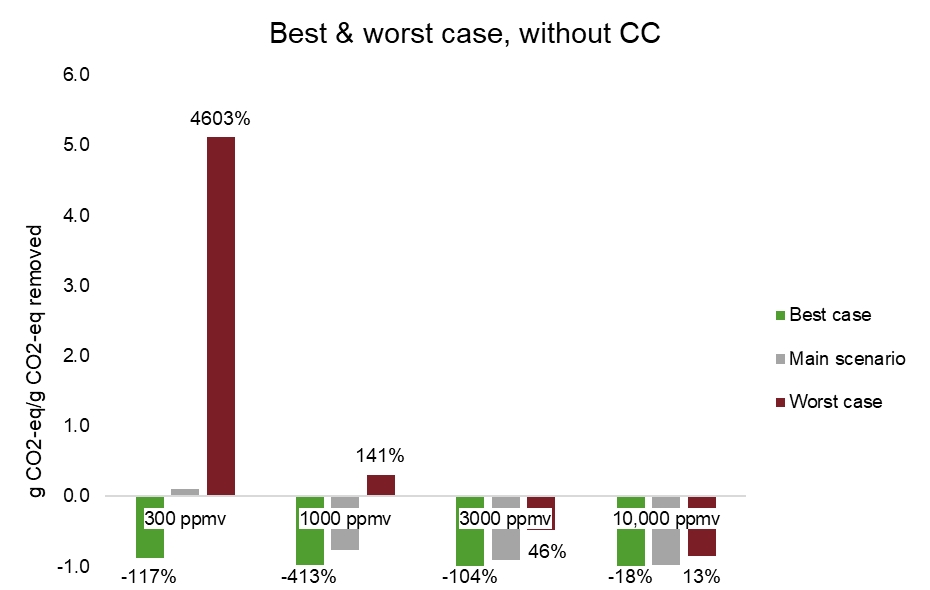


Figure S10. Net climate effect of CH_4_ conversion for the four modelled CH_4_ concentrations. The data labels show the relative increase/decrease compared to the main scenario (GWP100 and European mix).


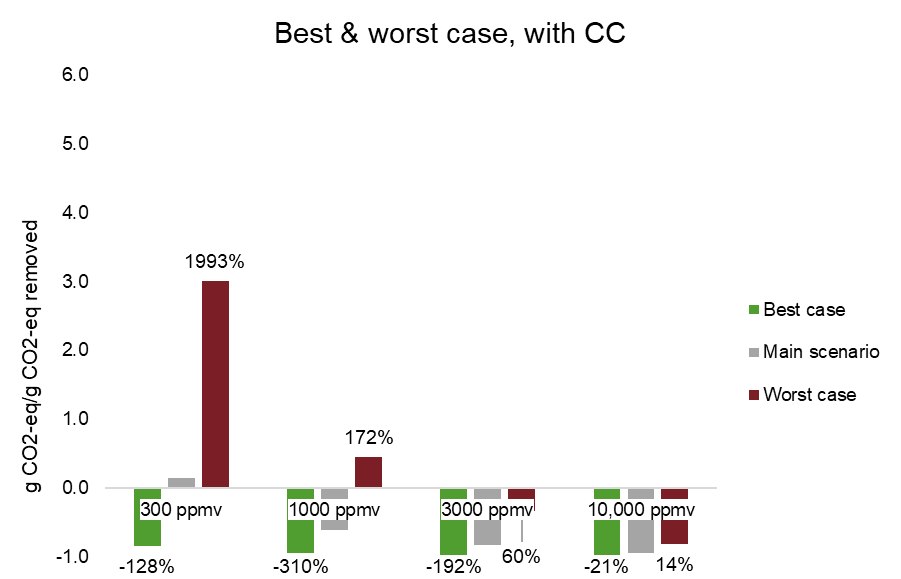


Figure S11. Net climate effect of co-removal for the four modelled CH_4_ concentrations. The data labels show the relative increase/decrease compared to the main scenario (GWP100 and European mix).

# Part B: Process modelling

The following modifications were made to the process model for CH_4_ conversion presented in Sirigina et al. (2023)

- The gas compositions and the volumetric flow rates corresponding to the three scenarios were considered.
- The conversion of CH_4_ in the reactor was set to 95%.
- Adiabatic conditions were assumed in the reactor.
- a MEA based absorption process for CO_2_ capture was considered for higher CO_2_ concentrations in contrast to amine-based adsorption at lower CO_2_ concentrations.


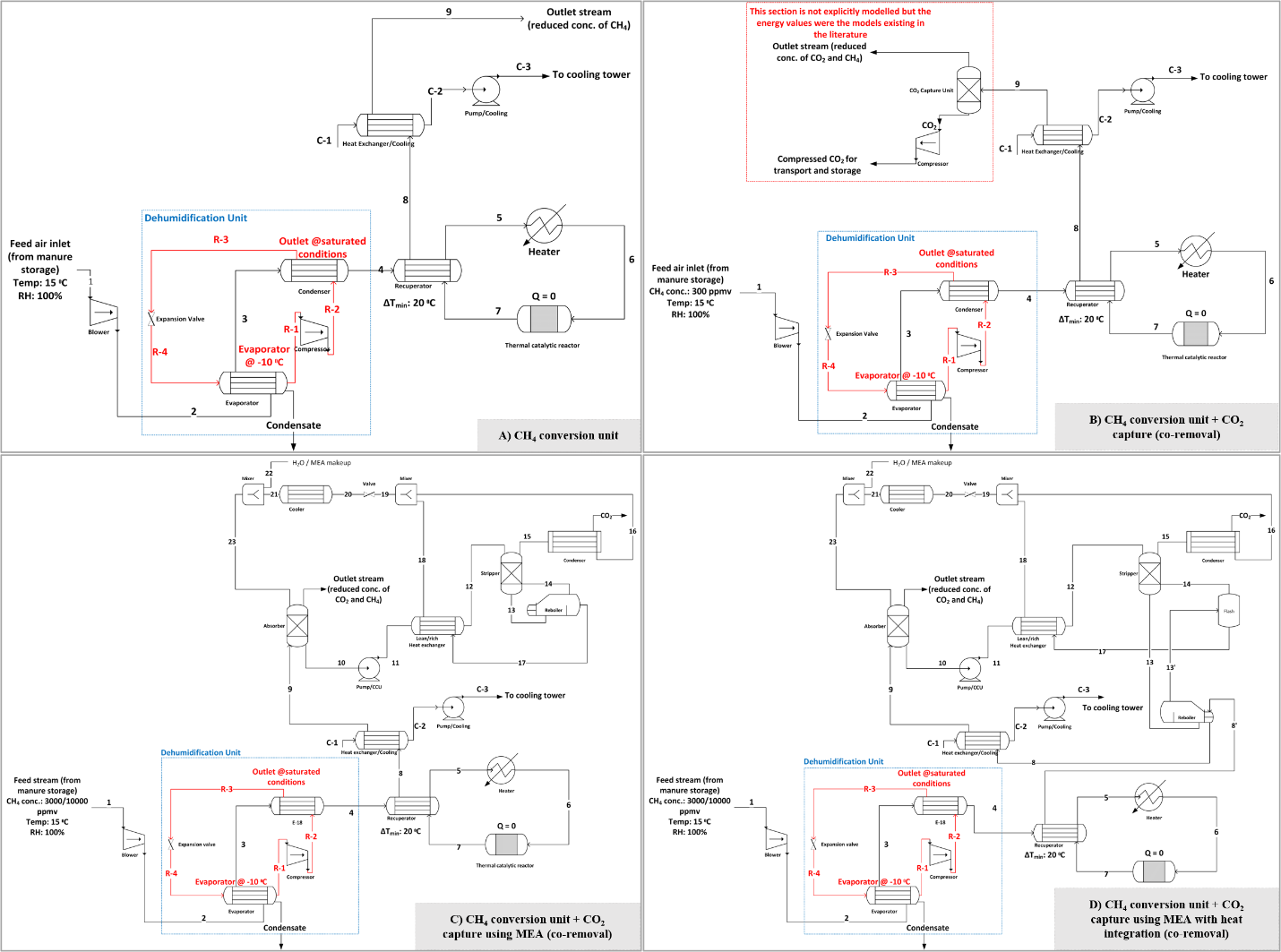


Figure S12. Process schematics for different cases considered in this study: A) CH_4_ conversion unit, B) CH_4_ Conversion unit with CO_2_ capture for the case with 300 ppmv inlet CH_4_ concentration (co-removal), C) CH_4_ conversion with CO_2_ capture using MEA absorption (co-removal) for 3000 ppmv, and 10,000 ppmv CH_4_ concentrations, and D) Includes heat integration between CH_4_ conversion and CO2 capture for cases with 3000 ppmv and 10,000 ppmv CH_4_ concentrations.

## Stream data

*Table S5. Stream data at process boundaries for the case with 300 ppmv CH_4_ concentration (adapted from Sirigina et al., 2023)*

| Boundary Stream | Volumetric flow (cum/hr) | Mass flow (kg/hr) | Temperature (K) | Pressure (bar) | CO_2_ mol% | CH_4_ mol% | H_2_O mol% | Rem. mol% |
| --- | --- | --- | --- | --- | --- | --- | --- | --- |
| 1 | 6890.00 | 8384.03 | 288.15 | 1.01 | 0.06 | 0.03 | 1.01 | 98.89 |
| Condensate | 0.02 | 25.35 | 274.15 | 1.24 | 0.00 | 0.00 | 100.00 | 0.00 |
| 9 | 6627.29 | 8358.68 | 293.15 | 1.07 | 0.09 | 0.00 | 0.59 | 99.32 |

Table S6. Stream data at process boundaries for the case with 1000 ppmv CH_4_ concentration

| Boundary Stream | Volumetric flow (cum/hr) | Mass flow (kg/hr) | Temperature (K) | Pressure (bar) | CO_2_ mol% | CH_4_ mol% | H_2_O mol% | Rem. mol% |
| --- | --- | --- | --- | --- | --- | --- | --- | --- |
| 1 | 2067.00 | 2514.69 | 288.15 | 1.01 | 0.11 | 0.10 | 1.02 | 98.78 |
| Condensate | 0.01 | 7.45 | 274.15 | 1.22 | 0.00 | 0.00 | 100.00 | 0.00 |
| 9 | 1988.42 | 2507.35 | 293.15 | 1.07 | 0.20 | 0.01 | 0.74 | 99.05 |

*Table S7. Stream data at process boundaries for the case with 3000 ppmv CH_4_ concentration*

| Boundary Stream | Volumetric flow (cum/hr) | Mass flow (kg/hr) | Temperature (K) | Pressure (bar) | CO_2_ mol% | CH_4_ mol% | H_2_O mol% | Rem. mol% |
| --- | --- | --- | --- | --- | --- | --- | --- | --- |
| 1 | 689.00 | 838.16 | 288.15 | 1.01 | 0.24 | 0.30 | 1.01 | 98.44 |
| Condensate | 0.00 | 2.46 | 274.15 | 1.22 | 0.00 | 0.00 | 100.00 | 0.00 |
| Outlet stream | 720.07 | 839.64 | 298.423 | 1.01 | 0.09 | 0.01 | 2.91 | 96.99 |
| CO_2_ | 1.77 | 5.71 | 308.15 | 1.90 | 96.80 | 0.00 | 3.03 | 0.18 |
| H_2_O/MEA Makeup | 0.01 | 9.64 | 300 | 1.03 | 0.00 | 0.00 | 99.96 | 0.04 |

*Table S8. Stream data at process boundaries for the case with 10,000 ppmv CH_4_ concentration*

| Boundary Stream | Volumetric flow (cum/hr) | Mass flow (kg/hr) | Temperature (K) | Pressure (bar) | CO_2_ mol% | CH_4_ mol% | H_2_O mol% | Rem. mol% |
| --- | --- | --- | --- | --- | --- | --- | --- | --- |
| 1 | 207.00 | 251.64 | 288.15 | 1.01 | 0.71 | 1.00 | 1.03 | 97.27 |
| Condensate | 0.00 | 0.76 | 274.15 | 1.22 | 0.00 | 0.00 | 100.00 | 0.00 |
| Outlet stream | 228.64 | 252.43 | 313.91 | 1.03 | 0.18 | 0.05 | 6.85 | 92.93 |
| CO_2_ | 1.76 | 5.87 | 308.15 | 1.96 | 96.99 | 0.00 | 2.95 | 0.07 |
| H_2_O/MEA Makeup | 0.01 | 7.00 | 313.50 | 1.03 | 0.00 | 0.00 | 100.00 | 0.00 |

*Table S9. The heat transfer coefficient and material data used in the CH_4_ conversion unit.*

| Equipment | Heat transfer coefficient (W / m^2^ K) | Material |
| --- | --- | --- |
| Evaporator | 138 | 304 Stainless steel |
| Condenser | 122 (Condensing refrigerant / Vapor)  30 (Vapor refrigerant /Vapor) | 304 Stainless steel |
| Recuperator | 30 | 304 Stainless steel |
| Heat exchanger – Cooling | 170 | 304 Stainless steel |

*Table S10. The heat transfer coefficient and material data used in the CO_2_ Capture unit (only for the 3000 ppmv and 10,000 ppmv CH_4_ cases)*

| Equipment | Heat transfer coefficient (W / m^2^ K) | Material |
| --- | --- | --- |
| Rich-lean heat exchanger | 500 | 316 Stainless steel |
| Cooler | 204 | 316 Stainless steel |
| Reboiler (for heat integration between CH_4_ conversion and CO_2_ capture) | 100 | 316 Stainless steel |

## Aspen output: Energy and material demand for the treatment process

The results of energy demand for the three scenarios are presented in Table S11. The presented data includes both CH_4_ conversion and co-removal (of methane and carbon dioxide) for the scenarios.

The energy demand presented in the table includes process heat integration between the CH_4_ conversion and CO_2_ capture units. The specific reboiler duty is 7.3 GJ/t-CO_2_ for the 10,000 ppmv CH_4_ case and 8.3 GJ/t-CO_2_ for the 3000 ppmv CH_4_ case. The heat integration is maximum at a 10,000 ppmv CH_4_ concentration with all the energy requirements of the reboiler being met by waste heat from CH_4_ conversion. Around 18% of reboiler duty was met by waste heat from the CH_4_ conversion unit for the case with 3000 ppmv of CH_4_ concentration. Heat integration was not possible for the cases with 300 ppmv and 1000 ppmv due to the low quality of the waste heat stream from the CH_4_ conversion unit.

Table S11. Energy demand (GJ/tonne CO­_2_-eq mitigated) for the three manure storage scenarios. The energy demand for 3000 ppmv and 10,000 ppmv CH_4_ concentrations includes process heat integration. CH_4_ conversion is denoted as Scenario A and Co-removal as Scenario B to decrease cluttering due to limited space.

| Concentration | 300 ppmv | | | 1000 ppmv | | | 3000 ppmv | | | 10,000 ppmv | | |
| --- | --- | --- | --- | --- | --- | --- | --- | --- | --- | --- | --- | --- |
| Scenario | **A** | **B** | **A** | | **B** | **A** | | **B** | **A** | | **B** |  |
| Blower | 6.04 | 4.49 | 1.64 | | 1.33 | 0.55 | | 0.47 | 0.16 | | 0.14 |  |
| Dehumidifier | 4.56 | 3.39 | 1.30 | | 1.05 | 0.43 | | 0.37 | 0.13 | | 0.11 |  |
| Heater | 3.42 | 2.55 | 0.00 | | 0.00 | 0.00 | | 0.00 | 0.00 | | 0.00 |  |
| Cooler Pump | 0.02 | 0.01 | 0.01 | | 0.00 | 0.00 | | 0.00 | 0.00 | | 0.00 |  |
| CO_2_ Capture Unit | - | 2.83 | - | | 2.08 | - | | 1.01 | - | | 0.04 |  |
| Total | **14.03** | **13.27** | **2.94** | | **4.47** | **0.98** | | **1.85** | **0.30** | | **0.30** |  |

The material requirement for CH_4_ conversion and CO_2_ capture is shown in Table S12. As described above, the CO_2_ capture technology differs with inlet CH_4_ concentration; an adsorption-based capture technology was considered for 300 ppmv, whereas an absorption-based process was considered for 3000 ppmv and 1% inlet CH_4_ concentrations. The catalyst estimation for CH_4_ conversion was based on the kinetic model presented in Alyani and Smith (2016). The catalyst amount was obtained assuming 95% conversion in the reactor. The amount of adsorbent for CO_2_ capture at 300 ppmv was based on data presented in Sabatino et al. (2021). For CO_2_ capture by MEA absorption, the makeup corresponding to the loss of MEA through the gas stream from the top of absorber is presented.

Table S12. Material requirements for CH_4_ conversion and CO_2_ capture.

| Material | 300 ppmv | | 1000 ppmv | | 3000 ppmv | | 10 000 ppmv | |
| --- | --- | --- | --- | --- | --- | --- | --- | --- |
| Catalyst for CH_4_ conversion  (6.5 wt% Pd/Al_2_O_3_) | 46755 g | 16064 g | | 7098 g | | 3966 g | |  |
| Adsorbent/absorbent  for CO_2_ capture | 47124 g | 31843 g | | 11 g/hr | | 8.4 g/hr | |  |

## Dimensioning of plant

Process components were sized automatically in Aspen Plus in accordance with the Table S13 trough Table S16.

Table S13. Sizing of equipment from the process model for the case with 300 ppm CH_4_.

| Unit | Specification (kW) | Equipment Weight** (kg) | Installed weight*** (kg) | Other installations |
| --- | --- | --- | --- | --- |
| Inlet blower (Capacity) | 56.47 | 3632.00 | 12794.17 | 9162.17 |
| Dehumidifier - Evaporator (Duty) | 107.35 | 908.00 | 2891.98 | 1983.98 |
| Dehumidifier - Condenser (Duty) | 149.96 | 1816.00 | 4436.03 | 2620.03 |
| Dehumidifier - Compressor (Capacity) | 42.62 | 6174.40 | 11474.40 | 5300.00 |
| Recuperator - Heat exchanger (Duty) | 606.67 | 19295.00 | 32031.52 | 12736.52 |
| Heater (Duty) | 32.02 | 444.92 | 7387.03 | 6942.11 |
| Reactor (Volume of reactor*) | 0.03 | 251.37 | 402.19 | 150.82 |
| Cooler - Heat exchanger (Duty) | 155.62 | 1180.40 | 4434.22 | 3253.82 |
| Pump (capacity) | 0.16 | 81.72 | 1559.04 | 1477.32 |
| Sum | 1150.87 | 33783.81 | 77410.58 | 43626.77 |

Table S14. Sizing of equipment from the process model for the case with 1000 ppm CH_4_.

| Unit | Specification (kW) | Equipment Weight** (kg) | Installed weight*** (kg) | Other installations |
| --- | --- | --- | --- | --- |
| Inlet blower (Capacity) | 15 | 2905.6 | 9711 | 6805 |
| Dehumidifier - Evaporator (Duty) | 31 | 354 | 1904 | 1550 |
| Dehumidifier - Condenser (Duty) | 43 | 636 | 2621 | 1986 |
| Dehumidifier - Compressor (Capacity) | 12 | 5766 | 10865 | 5099 |
| Recuperator - Heat exchanger (Duty) | 194 | 4767 | 11940 | 7173 |
| Heater (Duty) | 0 | 0 | 0 | 0 |
| Reactor (Volume of reactor*) | 0.0108 | 80 | 128 | 48 |
| Cooler - Heat exchanger (Duty) | 48 | 400 | 2788 | 2388 |
| Pump (capacity) | 0.05 | 77 | 1422 | 1345 |
| Sum | 342 | 14985 | 41378 | 26394 |

Table S15. Sizing of equipment from the process model for the case with 3000 ppm CH_4_.

| Unit | Specification (kW) | Equipment Weight** (kg) | Installed weight*** (kg) | Other installations |
| --- | --- | --- | --- | --- |
| Inlet blower (Capacity) | 5 | 2679 | 8360 | 5681.36 |
| Dehumidifier - Evaporator (Duty) | 10 | 213 | 1597 | 1383.34 |
| Dehumidifier - Condenser (Duty) | 14 | 277 | 1835 | 1557.67 |
| Dehumidifier - Compressor (Capacity) | 4 | 5584 | 10583 | 4998.99 |
| Recuperator - Heat exchanger (Duty) | 65 | 1044 | 8225 | 7180.46 |
| Heater (Duty) | 0 | 0 | 0 | 0.00 |
| Reactor (Volume of reactor*) | 0.00 | 38 | 61 | 22.90 |
| Cooler - Heat exchanger (Duty) | 28 | 232 | 2531 | 2299.51 |
| Pump (capacity) | 0.03 | 77 | 1111 | 1034.21 |
| Sum | 126.64 | 10144.20 | 34302.65 | 24158.44 |
| CCU - Absorber | - | 7264.00 | 11890.71 | 4627 |
| CCU - Stripper | - | 2406.20 | 6595.71 | 4190 |
| CCU - Rich/lean Heat Exchanger | 44.25 | 222.46 | 1497.75 | 1275 |
| CCU - Stripper Reboiler flash vessel | 13.00 | 862.60 | 4554.98 | 3692 |
| CCU - Stripper Condenser flash vessel | - | 862.60 | 4471.90 | 3609 |
| CCU - Cooler | 0.40 | 81.72 | 883.48 | 802 |
| CCU - Pump | 0.01 | 77.18 | 760.45 | 683 |

Table S16. Sizing of equipment from the process model for the case with 10,000 ppm CH_4_, the largest concentration requiring the smallest plant.

| Unit | Specification (kW) | Equipment Weight** (kg) | Installed weight*** (kg) | Other installations |
| --- | --- | --- | --- | --- |
| Inlet blower (Capacity) | 2 | 2588 | 6973 | 4385 |
| Dehumidifier - Evaporator (Duty) | 3 | 132 | 1270 | 1139 |
| Dehumidifier - Condenser (Duty) | 4 | 204 | 1595 | 1391 |
| Dehumidifier - Compressor (Capacity) | 1 | 5448 | 10319 | 4871 |
| Recuperator - Heat exchanger (Duty) | 20 | 209 | 4912 | 4703 |
| Heater (Duty) | 0 | 0 | 0 | 0 |
| Reactor (Volume of reactor*) | 0.00 | 21 | 34 | 13 |
| Cooler - Heat exchanger (Duty) | 21 | 182 | 2396 | 2214 |
| Pump (capacity) | 0.02 | 77 | 1111 | 1034 |
| Sum | 51.21 | 8860.38 | 28610.12 | 19749.74 |
| CCU - Absorber | - | 4358 | 8897 | 4539 |
| CCU - Stripper | - | 3042 | 7328 | 4287 |
| CCU - Stripper Reboiler - HX | 11.80 | 132 | 2492 |  |
| CCU - Rich/lean Heat Exchanger | - | 132 | 1180 | 1048 |
| CCU - Stripper Reboiler flash vessel | 0.00 | 863 | 4493 | 3631 |
| CCU - Stripper Condenser flash vessel | - | 863 | 4305 | 3443 |
| CCU - Cooler | - | 132 | 947 | 815 |
| CCU - Pump | 0.02 | 77.18 | 932.06 | 854.88 |

3000 & 10,000 ppmv are high enough concentrations of CH_4_ that the amount of heat released from the CH_4_ oxidation gives a temperature high enough for the catalyst to operate without preheating.

## Dimensioning of the reactor

The design of the reactor under adiabatic conditions is carried out by considering the reactor as a pressure vessel. The minimum wall thickness under the design conditions for 3 cases were estimated using the design equations presented in Table 12-10 by Peter et al. (2003). With an outer diameter of 0.0508 m, the inner diameter is estimated to be 0.0194 m. Considering a tube length of 3 m for the case with a 1% vol CH_4_ concentration, the amount of stainless steel 304 required for the pressure vessel was estimated. The density of the stainless steel 304 was considered to be 8000 kg/m^3^. For the case with 3000 ppmv of CH_4_ concentration, the same diameter values were assumed with a tube length of 4.1 m. The weight of two heads and nozzles was not factored into the calculation because it is observed that the weight of the reactor is insignificant compared to other process equipment.

## CO_2_ Capture by absorption

The CO_2_ capture for the cases with 3000 ppmv and 10,000 ppmv CH_4_ was considered through monoethanolamine (MEA) absorption. The modelling of CO_2_ capture done by aqueous MEA solution was carried out in Aspen Plus. The CO_2_ capture model was then coupled to the CH_4_ conversion unit. MEA absorption is well studied and among the most common technologies for post combustion CO_2_ capture (Wang *et al.* 2023, Madeddu *et al.* 2018). However, the technology was not well researched for CO_2_ capture from ambient air. Kiani et al. (2020) presented a study on the application of MEA absorption for the CO_2_ capture from ambient air. For the base case design (with a 50% capture rate), the reboiler duty and total electricity consumption were reported as 10.7 GJ/tCO_2_ and 1.452 MWh/tCO_2_. Although the energy demand at lower capture rates was reported to be low, a higher capital cost was reported due to the need for processing large amounts of air, suggesting the use of an alternative design for the absorber. In a comparative assessment of different direct air capture technologies, Sabatino et al. (2021) reported reboiler duty for CO_2_ capture by MEA absorption to be between 18.01 GJ/tCO_2_ and 48.16 GJ/tCO_2_ for the cases with minimum energy demand and maximum productivity, respectively. Both studies reveal a greater requirement of low-grade heat at higher CO_2_ capture rates. Therefore, in the current study, for the case with the lowest CH_4_ concentrations (resulting in a low CO_2_ concentration to the capture unit), CO_2_ capture using a solid sorbent technology with a much lower energy demand was considered. However, for the other cases with higher CH_4_ concentrations (resulting in a higher CO_2_ concentration to the capture unit), CO_2_ capture by MEA absorption was considered.

The modelling of CO_2_ capture in aqueous MEA solution was carried out following the standard process design for MEA absorption processes in post combustion CO_2_ capture. The inlet flue stream with CO_2_ flows countercurrent to the lean solution and CO_2_ is chemically absorbed into the solution. The CO_2_ rich stream from the bottom of the absorber is pumped to the stripper pressure and is later heated in the lean-rich heat exchanger. The rich solution is stripped of CO_2_ in the stripper unit and the hot lean solution flows through the lean-rich heat exchanger, exchanging heat with the cold rich stream from the absorber. The cold lean solution flows to the top of the absorber, completing the cycle. The gaseous stream from the top of stripper is sent to the condenser, where the water present in the stream is condensed. The concentrated CO_2_ stream from the condenser is sent for compression for further transport and permanent storage. The values for compression, transport, and storage were taken from the literature. The process schematics for CO_2_ capture by MEA absorption integrated to CH_4_ conversion is shown in Figure S12. A rate-based model for CO_2_ capture was developed in Aspen Plus to simulate the absorption process. The property evaluation was based on the ENTRL method present in Aspen Plus v12. Absorption and stripping were simulated using RadFrac models present in Aspen Plus. A structured packing type, Sulzer Mellapak 252 Y, was chosen as the packing type for both the columns. The correlations to estimate parameters corresponding to material and energy transfer for the packing type are readily available in Aspen Plus. The values for the rate-based model setup in Aspen Plus were obtained from Madeddu et al. (2018). The main assumptions behind the model are shown in Table S17 and Table S18. The modelling of CO_2_ capture and its integration with the CH_4_ conversion unit was performed separately for both the cases because of differences in concentrations, flow rates, and configuration. Complete heat integration was possible in the case with 10,000 ppmv CH_4_ concentration, with the waste heat from CH_4_ conversion unit utilized in the reboiler for CO_2_ regeneration. In the case with 3000 ppmv of CH_4_ concentration, about 18% of waste heat from CH_4_ conversion could be utilized in the reboiler.

Table S17. Modelling assumptions and results of CO_2_ capture by MEA absorption for the case with 10,000 ppmv CH_4_ concentration.

|  | Parameter | Value |
| --- | --- | --- |
| Inlet CO_2_ | Volume fraction | 1.7% |
| Lean solvent characterization | Temperature | 313.5 K |
|  | Pressure | 1 atm |
|  | CO_2_ loading (CO_2_ mole fraction / MEA mole fraction) | 0.3 |
| Absorber | Stages | 20 |
|  | Height | 9 m |
|  | Diameter | 0.25 m |
| Stripper | Pressure | 1.96 bar |
|  | Stages | 15 |
|  | Height | 3 m |
|  | Diameter (max.) | 0.077 m |
| Reboiler | Temperature | 393 K |
|  | Duty | 11.8 kW |
| Condenser | Temperature | 308.15 K |
| Lean-rich heat exchanger | ∆T_min_ | 10 K |
| Performance | CO_2_ capture rate | 89% |
|  | CO_2_ purity | 97% |

Table S18. Modelling assumptions and results of CO_2_ capture by MEA absorption for the case with 3000 ppmv CH_4_ concentration.

|  | Parameter | Value |
| --- | --- | --- |
| Inlet CO_2_ | Volume fraction | 5290 ppmv |
| Lean solvent characterization | Temperature | 300 K |
|  | Pressure | ~1 atm |
|  | CO_2_ loading (CO_2_ mole fraction / MEA mole fraction) | 0.281 |
| Absorber | Stages | 20 |
|  | Height | 11 m |
|  | Diameter | 0.27 m |
| Stripper | Pressure | 1.9 bar |
|  | Stages | 20 |
|  | Height | 3 m |
|  | Diameter | 0.089 m |
| Reboiler | Temperature | 394 K |
|  | Duty | 13 kW |
| Condenser | Temperature | 308.15 K |
| Lean-rich heat exchanger | ∆T_min_ | 10 K |
| Performance | CO_2_ capture rate | 83% |
|  | CO_2_ purity | 97% |

For the 3000 ppmv CH_4_ concentration case, it is observed that about 18% of reboiler energy demand was met using the thermal energy from the CH_4_ conversion unit.

# References

Abernethy, S., Kessler, M.I. & Jackson, R.B. (2023). Assessing the potential benefits of methane oxidation technologies using a concentration-based framework. Environmental Research Letters, 18(9), 094064. h
<ttps://doi.org/10.1088/1748-9326/acf603>

Bastos, J.L.V., Eleonora; Muntean, Marilena; Duerr, Marlene; Kona, Albana; Bertoldi, Paolo (2020). GHG Emission Factors for Electricity Consumption. http://data.europa.eu/89h/919df040-0252-4e4e-ad82-c054896e1641

Cogen Europe (2017). EU Primary *Energy Factor for Electricity: key to ensure well-informed choices, achieve real energy savings and reduce consumer energy bills*. https://www.cogeneurope.eu/wp-content/uploads/2024/07/2017_05_26_COGEN_Europe_Primary_Energy_Factor_Position.pdf

Fjelsted, L., Scheutz, C., Christensen, A.G., Larsen J.E., Kjeldsen, P. (2020). Biofiltration of diluted landfill gas in an active loaded open-bed compost filter. Waste management 103 , 1-11 <https://doi.org/10.1016/j.wasman.2019.12.005>

Gålfalk, M., Nilsson Påledal, S., Yngvesson, J., & Bastviken, D. (2024). Measurements of Methane Emissions from a Biofertilizer Storage Tank Using Ground-Based Hyperspectral Imaging and Flux. Environmental Science & Technology, 58(8), 3766-3775. <https://doi.org/10.1021/acs.est.3c06810>

IPCC (2019). *2019 Refinement to the 2006 IPCC Guidelines for National Greenhouse Gas Inventories. Chapter 10: Emissions from Livestock and Manure Management*. https://www.ipcc-nggip.iges.or.jp/public/2006gl/pdf/4_Volume4/V4_10_Ch10_Livestock.pdf

IPCC (2021). Assessment report 6. Chapter 7: The Earth’s Energy Budget, Climate Feedbacks, and Climate Sensitivity

Kiani, A., Jiang, K., & Feron, P. (2020). Techno-Economic Assessment for CO2 Capture From Air Using a Conventional Liquid-Based Absorption Process [Original Research]. Frontiers in Energy Research, 8. <https://doi.org/10.3389/fenrg.2020.00092>

Jia, W., Jia, P., Gu, L., Ren, L., Zhang, Y., Chen, H., Wu, X., Feng, W., Cai, J. (2025). Quantification of methane emissions from typical natural gas stations using on-site measurement technology. *Journal of Pipeline Science and Engineering, 5*(2), 100229. <https://doi.org/10.1016/j.jpse.2024.100229>

Madeddu, C., Errico, M., & Baratti, R. (2018). *CO_2_ capture by reactive absorption-stripping: modeling, analysis and design*. Springer.

Melse, R. W., & van der Werf, A. W. (2005). Biofiltration for Mitigation of Methane Emission from Animal Husbandry. Environmental Science & Technology, 39 (14), 5460-5468 <https://doi.org/10.1021/es048048q>

NOAA. (2024). *Trends in Atmospheric Methane (CH_4_).* National Oceanic & Atmospheric Administration Global Monitoring Laboratory. <https://gml.noaa.gov/ccgg/trends_ch4/> (Accessed: 2025-03-01)

Pazhanivelan, S., Sudarmanian, N. S., Geethalakshmi, V., Deiveegan, M., Ragunath, K., Sivamurugan, A. P., & Shanmugapriya, P. (2024). Assessing Methane Emissions from Rice Fields in Large Irrigation Projects Using Satellite-Derived Land Surface Temperature and Agronomic Flooding: A Spatial Analysis. *Agriculture*, *14*(3), 496. <https://doi.org/10.3390/agriculture14030496>

Poblete, I.B.S., Araujo, O.d.Q.F. & de Medeiros, J.L. (2020). Dynamic analysis of 804 sustainable biogas-combined-cycle plant: Time-varying demand and 805 bioenergy with carbon capture and storage. *Renewable and Sustainable* 806 *Energy Reviews,* 131. <https://doi.org/10.1016/j.rser.2020.109997>

Quaschning, V. (2022). Specific Carbon Dioxide Emissions of Various Fuels. https://www.volker-quaschning.de/datserv/CO2-spez/index_e.php [2023-04-02]

Sabatino, F., Grimm, A., Gallucci, F., van Sint Annaland, M., Kramer, G. J., & Gazzani, M. (2021). A comparative energy and costs assessment and optimization for direct air capture technologies. Joule, 5(8), 2047-2076. <https://doi.org/10.1016/j.joule.2021.05.023>

Sandgren, A. & Nilsson, J. (2021). Emissionsfaktor för nordisk elmix med hänsyn till import och export - Utredning av lämplig systemgräns för elmix samt beräkning av det nordiska elsystemets klimatpåverkan. miljöinstitutet, I.S. https://www.ivl.se/download/18.556fc7e17c75c84933f392/1635759400558/FULLTEXT01.pdf [2022-08-01]

Sirigina, D.S.S.S., Goel, A. & Nazir, S.M. (2023). Process concepts and analysis for co-removing methane and carbon dioxide from the atmosphere. Scientific Reports, 13(1), 17290. <https://doi.org/10.1038/s41598-023-44582-w>

Sweco (2021). Carbon intensity and primary energy factors. (300031634). Energimyndigheten.

Tabase, R.K., Næss, G., Larring, Y. (2023). Ammonia and methane emissions from small herd cattle buildings in a cold climate. *Science of the total environment, 903*, 166046

Terlouw, T., Treyer, K., Bauer, C. & Mazzotti, M. (2021). Life Cycle Assessment of Direct Air Carbon Capture and Storage with Low-Carbon Energy Sources. *Environmental Science & Technology,* 55(16), 11397-11411. https://doi.org/10.1021/acs.est.1c03263

Ursueguía, D., Marín, P, Díaz, E., Ordóñez, S. (2021) A new strategy for upgrading ventilation air methane emissions combining adsorption and combustion in a lean-gas turbine.  *Journal of Natural Gas Science and Engineering*, *88*, 103808

Wang, M., Lawal, A., Stephenson, P., Sidders, J., & Ramshaw, C. (2011). Post-combustion CO2 capture with chemical absorption: A state-of-the-art review. *Chemical Engineering Research and Design*, *89*(9), 1609-1624. [https://doi.org/10.1016/j.cherd.2010.11.005](https://doi.org/https://doi.org/10.1016/j.cherd.2010.11.005)
